# Cholesterol Metabolism Regulated Nanoliposome Ameliorates Chemo/Photothermal Therapy Reversing CD8^+^ T Cell Exhaustion

**DOI:** 10.1002/EXP.20240123

**Published:** 2025-12-07

**Authors:** Panpan Xue, Tingjie Bai, Huilan Zhuang, Angelo H. All, Shuangqian Yan, Xuemei Zeng

**Affiliations:** ^1^ Key Laboratory of Microbial Pathogenesis and Interventions of Fujian Province University Biomedical Research Center of South China College of Life Sciences Fujian Normal University Fuzhou P. R. China; ^2^ Strait Institute of Flexible Electronics (SIFE Future Technologies) Fujian Normal University Fuzhou China; ^3^ Strait Laboratory of Flexible Electronics (SLoFE) Fuzhou China; ^4^ Hong Kong Baptist University Hong Kong SAR China

**Keywords:** checkpoint blockade, cholesterol metabolism, photothermal therapy, T cell exhaustion

## Abstract

Stimulating the immunogenic cell death (ICD) by chemical and photothermal agents triggers antitumor immune responses to tumor treatments. However, the abnormal cholesterol accumulation in cancer cells promotes tumor survival and metastasis, while the high concentration of cholesterol in the tumor microenvironment will result in T exhaustion. Herein, a cholesterol‐regulable nanoliposome (ictLipo) that encapsulated with IR806, tirapazamine, and cholesterol oxidase (ChOx) was fabricated to boost chemo/photothermal‐activated ICD and antitumor immune responses. Specifically, the released ChOx oxidizes cholesterol, accompanied by oxygen consumption and ATP reduction. Whereupon, tumoral anabatic hypoxia activates the toxicity of tirapazamine while ATP reduction strengthens photothermal therapy of the IR806 by suppression of HSP70. We found that this interactive strategy evokes robust ICD and reverses T‐cell exhaustion. When combined with the immune checkpoint blockade, ictLipo can efficiently restrain breast cancer metastasis in two tumor models. The interactive strategy sheds new light on the immunological facet of cholesterol metabolism and may provide a new therapeutic strategy against breast cancer.

## Graphical abstract

Our work addresses the challenge of treating triple‐negative breast cancer by developing a multifunctional nanoliposome, ictLipo. The goal was to enhance antitumor immunity by combining photothermal therapy and chemotherapy while simultaneously tackling two major roadblocks: immunosuppression from cholesterol in the tumor microenvironment and therapy resistance caused by heat shock proteins.

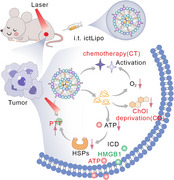

## 1 Introduction

Breast cancer was the most common cancer in 2022, with 2.26 million new cases, and triple‐negative breast cancer (TNBC) accounted for 15%–20% of these cases [1]. TNBC is notably more aggressive and invasive compared to other breast cancer subtypes, characterized by a high rate of metastasis and poor prognosis [1]. Current treatment strategies, such as mastectomy combined with chemotherapy and radiotherapy, often result in significant aesthetic damage, severe side effects, and limited therapeutic efficacy [2]. Encouragingly, cancer immunotherapy has emerged as a promising approach for TNBC treatment. Immune checkpoint inhibitors targeting programmed cell death protein 1 (PD‐1) and programmed death‐ligand 1 (PD‐L1) have been introduced in clinical settings for TNBC therapy [3, 4]. However, the benefits of immunotherapy are restricted to a small subset of patients, due to moderate immune responses and T‐cell exhaustion, which are driven by abnormal lipid metabolism and an immunosuppressive tumor microenvironment (TME) [5, 6].

Like colon and prostate cancers, breast cancer, including TNBC, exhibits abnormal cholesterol metabolism, that is to say, cholesterol accumulation in cancer cells and TME [7, 8, 9]. Functionally, as a main component of the lipid raft in the cell membrane, cholesterol and related metabolism affect the fate of both cancer cells and immune cells [10, 11]. Cholesterol accumulation in cancer cells can promote tumor proliferation, migration, and invasion. Specifically, cholesterol can mediate cell cycle protein Cyclin D1 and c‐Myc to facilitate cancer cell proliferation [12]. The cholesterol in lipid rafts may activate RAS‐MAPK pathways and epithelial‐mesenchymal transition to induce cell migration and invasion [13]. Besides, cholesterol accumulation in TME will lead to CD8^+^ T cell exhaustion in an ER‐stress‐XBP1‐dependent manner, which is responsible for the effector function loss of tumor‐infiltrating T cells [14]. Thus, cholesterol metabolism regulation or cholesterol deprivation in tumor cells and TME has emerged as an effective strategy for priming T cell functions and combating tumors [15, 16, 17, 18, 19].

Notably, induction of immunogenic cell death (ICD) is a powerful manner for rejuvenating the antitumor immune responses by chronic exposure of damage‐associated molecular patterns (DAMPs) in TME [20, 21]. Preclinical and clinical studies have proven that photothermal therapy (PTT) and chemotherapy (CT) are amenable to incite ICD, and the combined PTT/CT may have a better efficacy than the single one [22]. Under near‐infrared (NIR) laser illumination, hyperthermia that emanates from PTT can give rise to cell apoptosis and followed by DAMPs release. Unfortunately, hyperthermia can also elevate the expression of heat shock proteins (HSP) such as HSP70, which in turn hampers PTT efficacy and following immune response abduction [22]. Similarly, CT with the ability to provoke ICD by stimulating cell apoptosis or other death mechanisms [23]. There is no denying that CT has severe systemic side effects due to its poor tumor specificity [2, 23]. The concerted PTT/CT can overcome the above‐mentioned dilemma to some extent and enhance the tumor‐infiltrating of T cells, but the infiltrated T cells will still be exhausted by cholesterol in the TNBC TME. Therefore, the combination of PTT/CT and cholesterol modulation represents a promising strategy for the treatment of TNBC. A deeper understanding of the immunological mechanisms underlying this approach is essential for advancing research in both oncology and immunology.

**FIGURE 1:**
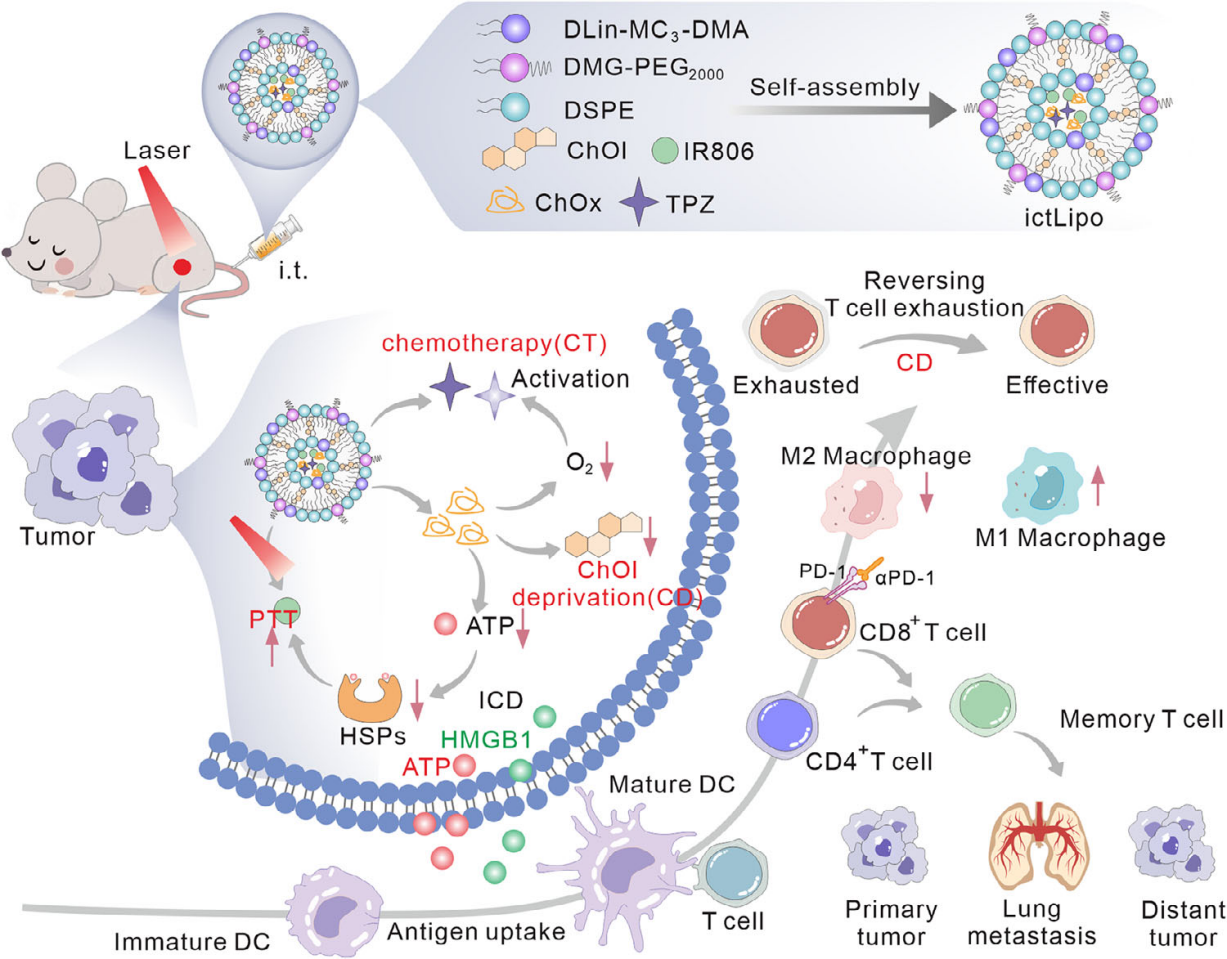
Schematic illustration of ictLipo nanoplatform for stimulating antitumor immune responses by cholesterol depletion‐mediated chemo/photothermal therapy (CD‐CT/PTT) and followed immunosuppressive TME reversion and T‐cell exhaustion inhibition. ictLipo nanoplatform is capable of inducing robust antitumor responses. Specifically, ictLipo can be concerted with the PD‐1 ICB for long‐term inhibiting the TNBC relapse and metastasis.

Motivated by the context described above, herein we propose to use a liposome‐based nanoplatform, named ictLipo, to induce robust ICD and reverse the immunosuppressive TME and exhausting T‐cell for TNBC treatment through cholesterol deprivation, PTT, and responsive CT in a concise and biosafety manner. The ictLipo consists of a nanosized liposome that is loaded with IR806, cholesterol oxidase (ChOx), and tirapazamine (TPZ) (Figure 1). After system injection, ictLipo can accumulate at the tumor sites. Under local NIR laser irradiation, the generated hyperthermia by IR806 not only induces tumor ICD but also enhances the activity of ChOx. As a type of oxidase, ChOx can oxide cholesterol to 4‐cholesten‐3‐one and H_2_O_2_, accompanied by oxygen consumption [24]. The hypoxia intensification by ChOx will activate the toxicity of TPZ [25] to realize cascade cholesterol depletion (CD) and thus exaggerate ICD. Meanwhile, the deprivation of cholesterol in TME and cancer cells may impede cell proliferation, metastasis, and remove the cholesterol metabolism‐associated immunosuppression. Interestingly, ChOx has no obvious effects on T cell viability. In addition, lipid raft destruction and cell cycle arrest by cholesterol depletion will inhibit ATP synthesis in cancer cells, which frustrates HSP70 synthesis and activation, thus eliminating the resistance of PTT. Such an interactive strategy based on the ictLipo nanoplatform is capable of inducing robust antitumor responses. Specifically, ictLipo can be concerted with the PD‐1 ICB for long‐term inhibiting the TNBC relapse and metastasis.

## 2 Results and Discussion

### 2.1 Preparation and Characterization of ictLipo

**FIGURE 2:**
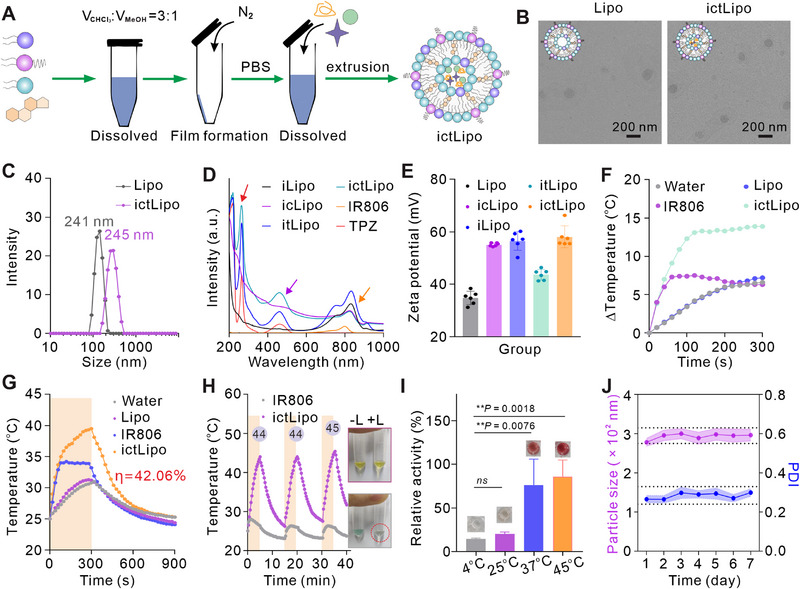
Preparation and characterization of ictLipo. (A) Schematic illustration of the synthetic procedures of ictLipo. (B) TEM images of Lipo and ictLipo. Scale bars are 200 nm. (C–E) Hydrodynamic diameter measurements (C), UV–vis spectra (D), and zeta potential (E) of ictLipo with various modifications, respectively. (F) NIR photothermal heating curves of ultrapure water, free IR806, Lipo, and ictLipo solutions exposed to 808 nm laser illumination for 5 min at 0.5 W/cm^2^. (G) Heating and cooling curves to calculate the photothermal conversion efficacy. (H) Photothermal stability and corresponding photos (inset) of free IR806 and ictLipo under NIR laser on/off cycles at 0.5 W/cm^2^. (I) The relative enzyme activity of ChOx and corresponding photos (inset) under various temperature conditions (*n* = 3). (J) Hydrodynamic size and PDI value of ictLipo dispersed in PBS supplemented with 10% FBS for 7 days storage. **p < 0.01, ****p < 0.0001, and ns: not significant (*p* > 0.05), analyzed by one‐way ANOVA, followed by Dunnett's multiple comparisons test. Data represent mean ± s.d.

The procedures to synthesize ictLipo are illustrated in Figure 2A. Briefly, a lipid mixture composed of Dlin‐MC_3_‐DMA, DSPE, DMG‐PEG_2000_, and cholesterol (with molar ratios of 50: 10: 1.5: 38.5, respectively) was dissolved in a chloroform–methanol mixture (3: 1 v/v). The solution was then subjected to nitrogen gas to evaporate the solvents, resulting in the formation of a thin lipid film on the surface of the tube. This film was hydrated with PBS buffer and subsequently extruded through an Avanti liposome extruder (with pore sizes of 800, 400, and 200 nm) for 30 cycles to produce liposome nanoparticles (Lipo) [25, 26]. For the synthesis of ictLipo, IR806, ChOx, and/or TPZ (in mass ratios of IR806 = 1: 5, ChOx = 1: 2, TPZ = 1: 40) were incorporated into the lipid solution before extrusion. The resulting solution was centrifuged twice to obtain purified ictLipo (Figure 2A). Transmission electron microscopy (TEM) images indicated that both Lipo and ictLipo possess a quasi‐spherical morphology of approximately 180 nm in diameter (Figure 2B). The hydrodynamic size of Lipo and ictLipo was 241 and 245 nm, respectively (Figure 2C). The existing hydrodynamic shell gave rise to a larger hydrodynamic size compared to the TEM results [27]. The characteristic peaks of ChOx (280 nm), TPZ (460 nm), and IR806 (806 nm) were visualized in the UV–vis absorption spectrum of ictLipo (Figure 2D). And the zeta potential changed significantly along with the modification process (Figure 2E). The above results verified the successful construction of ictLipo. The loading efficiency of TPZ and IR806 was calculated to be 79.69% and 93.53%, respectively, via the corresponding standard curves of UV–vis spectra (Figures S1 and S2). While the loading efficiency of ChOx was approximately 95%, which was determined by a BCA protein assay (Figure S3) and a nanodrop measurement (Figure S4).

Next, we investigated the photothermal performance of ictLipo. As shown in Figure 2F, Lipo exhibited the same temperature increase compared to water under 808 nm laser irradiation (0.5 W/cm^2^), suggesting that Lipo has a minimal photothermal effect. As a veteran photothermal agent, IR806 displayed a concentrate‐dependent temperature increase. However, the temperature of the IR806 solution only increased in the initial 2 min and decreased in further illumination, which may result from the photobleaching (Figure S5). By comparison, the temperature of ictLipo climbed 14°C and its photothermal conversion efficacy was 42.06% (Figure 2G and Figure S6). As can be seen from Figure 2H, ictLipo had excellent photothermal stability while IR806 presented an obvious change in color after laser illumination (inset Figure 2H), which may be due to a distinct configuration of IR806 in the lipid bilayer [28], reflecting the protection capacity of the nanoliposome to IR806. Together, the above results indicate that the as‐prepared ictLipo has good photothermal performance and shows promise in the application of PTT. We next investigated whether the elevated temperature affects the enzymatic activity of ChOx by a 4‐amino‐antipyrine colorimetric method [29]. Practically, ChOx can catalyze cholesterol to 4‐cholesten‐3‐one and H_2_O_2_ in the presence of oxygen. Meanwhile, the 4‐amino‐antipyrine will react with phenol in the presence of H_2_O_2_ and peroxidase, followed by the generation of red substances which possess an absorption peak at 500 nm. As shown in Figure 2I, we found that the enzymatic activity of ChOx elevated obviously along with increasing temperature, suggesting that ChOx and PTT may have a synergistic effect on cholesterol depletion and tumor therapy.

Colloidal stability is a prerequisite for further in vitro and in vivo applications of nanoplatforms. Thus, we investigated the colloidal stability of ictLipo in PBS and PBS supplemented with 10% FBS. According to Figure S7, the size and polydispersity index (PDI) of ictLipo displayed no obvious disturbance in PBS during storage for 7 days at 4°C. Similarly, in the PBS supplemented with 10% FBS for 7 days, the size and PDI of ictLipo range about 150 nm and 0.3, respectively (Figure 2J). The present results indicate that ictLipo has good colloidal stability during storage.

### 2.2 Cell Uptake and Cholesterol Depletion of ictLipo in 4T1 Cells

**FIGURE 3:**
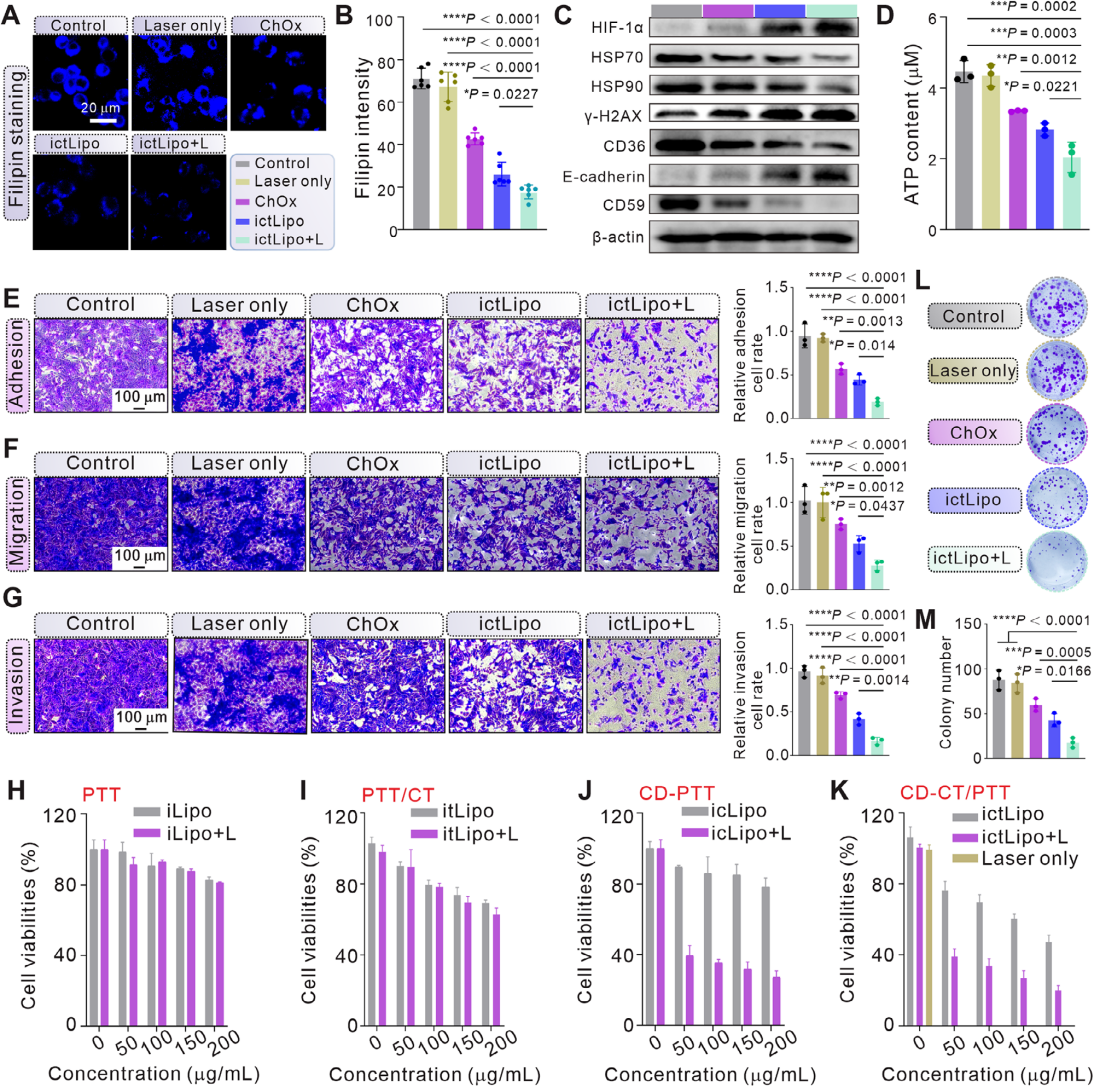
Cholesterol depletion can ameliorate the therapeutic efficacy of CT/PTT by ictLipo in vitro. (A,B) CLSM images (A) and corresponding statistical results (B) of 4T1 incubated with indicated formulas. Cholesterol was stained with Filipin. Scale bars are 20 µm. (C) The results of the western blotting assay from 4T1 received indicated treatments. (D) Comparison of intracellular ATP content after various treatments. (E–G) The representative images and statistical results of adhesion (E), migration (F), and invasion (G) assays, respectively (*n* = 3). (H–K) Cell viabilities of 4T1 cells incubated with various concentrations of iLipo (H), itLipo (I), icLipo (J), and ictLipo (K) with or without laser irradiation (*n* = 4). (L,M) The photos and statistical results of the colony assay (*n* = 3). *p < 0.05, **p < 0.01, ***p < 0.001, ****p < 0.0001, and ns: not significant (*p* > 0.05), analyzed by one‐way ANOVA, followed by Dunnett's multiple comparisons test. Data represent mean ± s.d.

Inspired by the outstanding photothermal performance of ictLipo and elevated ChOx enzyme activity at high temperatures, we further explored the biological applications of ictLipo in 4T1 cells. The cellular uptake behavior of ictLipo was first studied. The FITC labeled ictLipo (ictLipo‐FITC) was synthesized and incubated with 4T1 cells at various time points, and the fluorescent intensity was detected by flow cytometry (FCM). As shown in Figure S8, the evident fluorescent intensity was detected in 4T1 cells incubated with ictLipo for 1 h, and intensity increased along with the incubation time, indicating good cellular uptake ability of ictLipo in 4T1 cells. Subsequently, the in vitro cholesterol depletion ability of ictLipo was investigated. Filipin complex is a fluorescent dye that can specifically unite cholesterol [24]. Thus, cells that were treated with various treatments were stained with Filipin. According to CLSM images (Figures 3A,B), we found that the laser‐only group showed considerable blue fluorescence intensity compared to the control group, while ChOx‐treated cells displayed weaker blue fluorescence intensity, implying the cholesterol depletion ability of the ChOx. Specifically, cells treated with ictLipo showed lower blue signals than the ChOx‐treated cells. This may be directly attributed to the positively charged ictLipo (58.1 mV) being prone to being swallowed by cancer cells than the negatively charged ChOx (−7.94 mV) (Figure S9) [30]. Unsurprisingly, cells treated with ictLipo + L exhibited minimal blue signals, indicating the enhanced effect of PTT on ChOx enzymatic activity, thus expediting cholesterol depletion, which is consistent with the observation from Figure 2I that higher temperature boosts the activity of ChOx.

### 2.3 ictLipo Induces Cell Death and Inhibits Cell Adhesion and Migration

As mentioned above, ChOx catalyzes cholesterol and oxygen to 4‐cholesten‐3‐one and H_2_O_2_, thus may further aggravate the hypoxia of the cancer cells. Thus, hypoxia‐inducible factor 1α (HIF‐1α) was measured by western blot assay to evaluate the hypoxia situation after various treatments. As shown in Figure 3C, the expression of HIF‐1α in cells that were treated with ChOx, ictLipo, and ictLipo + L elevated 185.98%, 459.85%, and 496.50%, respectively, compared to the control group, implying the intensified hypoxia by cholesterol depletion, endowing the potential of the ChOx in activating the hypoxia‐responsive TPZ in cancer cells. Hyperthermia tends to induce the expression of heat shock proteins (HSPs) such as HSP70 and HSP90, which will attenuate the efficacy of PTT through the acquired thermoresistance [22]. Admittedly, both the process of expression and activation of HSPs are ATP‐involved. Thus, restricting ATP supply is an effective strategy to improve PTT [31]. Unsurprisingly, the laser‐only group did not alter the expression of related proteins, excluding the influence of laser illumination (Figure S10). Interestingly, we found that ChOx can destroy cellular ATP contents in a concentration‐dependent manner (Figure S11), which may stem from the ChOx can destroy the cellular lipid raft and inhibit cell proliferation. As illustrated in Figure 3D, laser laser‐only group exhibited no impact on ATP level compared to the control group, and ictLipo decreased ATP level in 4T1 cells, and its ATP depletion ability was enhanced under laser irradiation. The results are in good consistency with hyperthermia can boost the enzymatic activity of ChOx in Figure 2I. Predictably, ChOx and ictLipo indeed can suppress the expression of HSP70 and HSP90. It was worth noting that cells treated with ictLipo and laser excitation (ictLipo + L) had lower HSP expression. This may hinge on the enhanced cell cycle arrest of ictLipo under laser irradiation, which was confirmed by the high expression of γ‐H2AX (Figure 3C). Due to the important role of cholesterol in lipid metabolism, the expression of fatty acid transport protein CD36 was investigated. Interestingly, the ChOx, ictLipo, and ictLipo treatments decreased the CD36 expression, suggesting cholesterol depletion restrained fatty acid uptake. Similar results were discovered in mouse T lymphocytes CTLL2 cells (Figure S12). Although the specific mechanism is unknown due to complicated lipid metabolism, the decreased CD36 in CTLL2 is capable of achieving efficacious immunotherapy applications [32]. Together, the above results suggested that cholesterol depletion mediated by ictLipo + L induced hypoxia aggravation, ATP decreasing, cell cycle sluggishness, and HSP decreasing, which is beneficial to activating TPZ and enhancing PTT and antitumor immune responses.

It has been well‐documented that breast cancer cells require cholesterol to promote cellular growth, invasion, and migration. Therefore, we conducted experiments to characterize the impact of ictLipo on cell invasion and migration. According to Figure 3C and Figure S10, laser only and ChOx exhibited no obvious effects on the expression of E‐cadherin compared to the control group. By comparison, ictLipo elevated the E‐cadherin expression, and its effects were enhanced under laser illumination. In addition, the expression of lipid raft‐related CD59 was reduced by ictLipo in which the cholesterol‐enriched lipid raft is highly related to the metastasis process. These results suggest that ictLipo exhibits promise in tumor metastasis inhibition. Next, the cell scratch assay and adhesion assay confirmed the inhibition effect of ictLipo on cell healing (Figure S13) and adhesion (Figure 3E). As can be seen from Figures 3F,G, the as‐prepared ictLipo could inhibit cellular migration and invasion, respectively.

Afterward, the synergic effects of cholesterol deprivation (CD), CT, and PTT by ictLipo on cell killing were investigated. As shown in Figure 3H, IR806‐loaded liposome (iLipo) had minimal effects on cell viability even under laser irradiation, which may originate from the thermoresistance of the cancer cells and short‐term laser treatment (5 min). IR806/TPZ‐loaded liposome (itLipo) exhibited moderate cytotoxicity with or without laser illumination (Figure 3I), suggesting that TPZ has minimal toxicity to cells under normoxia. CD can improve the efficacy of PTT through the inhibition of HSPs, which was confirmed by the viability plummet of cancer cells that were treated with icLipo (IR806/ChOx‐encapsulated liposome) +L (Figure 3J). Specifically, ictLipo without laser treatment presented higher therapeutic efficacy than itLipo (Figure 3I), demonstrating the synergistic effect between ChOx and TPZ (Figure 3K). Under laser illumination, ictLipo significantly decreased the cell viability (Figure 3K and Figure S14). Specifically, ictLipo had no obvious effects on CTLL2 (Figure S15), which may be ascribed to CTLL2 having comparatively fallow lipid metabolism than cancer cells. In addition, the long‐term impact of ictLipo + L on cell viability was investigated by the cloning assay (Figures 3L,M). The colony number of ChOx, ictLipo, and ictLipo+L treatments decreased 32%, 52%, and 80%, respectively, which further verifies the outstanding CD‐CT/PTT efficacy of the ictLipo + L. All considered, these results suggest that CD by ChOx can facilitate the activation of TPZ and enhancement of PTT and thus achieve a valid combined CD‐CT/PTT.

### 2.4 ICD induced by ictLipo Plus Laser

**FIGURE 4:**
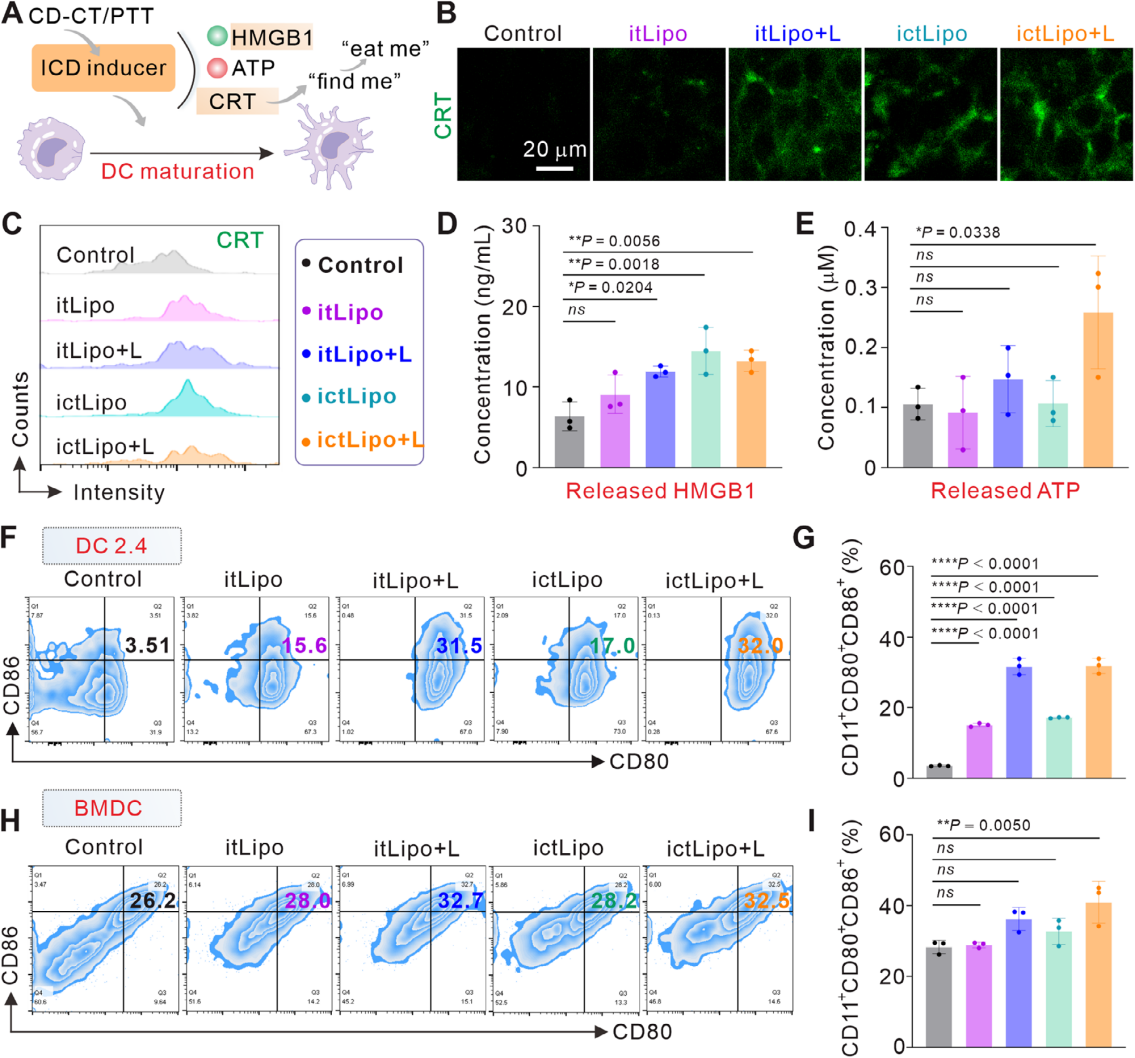
ictLipo induces ICD and in vitro immune responses. (A) Schematic of ICD and DC maturation induced by ictLipo. (B,C) CLSM images (B) and flow cytometric results (C) of CRT expression in 4T1 cells (*n* = 3). (D,E) HMGB1 (D) and ATP (E) contents in cellular supernatant after different treatments (*n* = 3). (F,G) Representative flow cytometry plots (F) and proportional statistics (G) indicating the efficacy of DC maturation (CD11^+^CD80^+^CD86^+^) upon indicated treatments in DC2.4 cells by a FCM analysis (*n* = 3). (H,I) Representative flow cytometry plots (H) and proportional statistics (I) indicating the efficacy of DC maturation (CD11^+^CD80^+^CD86^+^) upon indicated treatments in BMDC by a FCM analysis (*n* = 3). *p < 0.05, **p < 0.01, ****p < 0.0001, and ns: not significant (*p* > 0.05), analyzed by one‐way ANOVA, followed by Dunnett's multiple comparisons test. Data represent mean ± s.d.

It has been uncovered that PTT or CT can induce ICD, followed by the release of DAMPs to trigger the antitumor immune responses [22]. The excellent in vitro therapeutic efficacy of ictLipo impels us to explore the ICD induced by ictLipo in 4T1 cells. ICD is featured by “eat me” signals derived from calreticulin (CRT) exposure and “find me” signals derived from high mobility group box 1 (HMGB1) release and ATP secretion (Figure 4A) [33]. On the basis of immunofluorescent staining (Figure 4B) and flow cytometry analysis (Figure 4C), we found that itLipo‐treated cells exhibited grimy green fluorescent while itLipo + L and ictLipo groups showed bright green fluorescence signals. Notably, the ictLipo + L group displayed apparent high fluorescence intensity, indicating that the synergistic effect of CD‐CT/PTT by ictLipo is capable of evoking “eat me” signals. Additionally, the supernatant of cells after various treatments was collected, and the HMGB1 and ATP were detected. As shown in Figures 4D,E and Figures S16 and S17, ictLipo + L enhanced the HMGB1 release and ATP secretion compared to other groups, implying the “find me” signals were stimulated by the ictLipo + L. Together, the above results reflected that ictLipo + L is capable of inducing ICD. Next, the maturation of dendritic cells was studied in two parallel cell lines including DC2.4 and bone marrow‐derived dendritic cells (BMDC) (gated on CD11c^+^CD80^+^CD86^+^). As shown in Figures 4F,G and Figure S18, ictLipo + L induced about 32% maturation of DC2.4 cells. Meanwhile, ictLipo can also stimulate the BMDC maturation (Figures 4H,I). Accordingly, ictLipo + L is able to initiate ICD in the TNBC cell line and boost DC maturation in vitro, showing potential in immune therapy.

### 2.5 In Vivo Pharmacokinetics, Biodistribution, and CD‐CT/PTT Performance of ictLipo

The promising anticancer properties and encouraging immune activation of ictLipo observed in vitro prompted further investigation of its therapeutic efficacy in vivo. We first assessed the in vivo pharmacokinetics of ictLipo. Free IR806 (100 µg) and ictLipo (containing 100 µg IR806) were intravenously administered to 4T1 tumor‐bearing mice, followed by tracking using an IVIS imaging system. As depicted in Figures S19A,B, ictLipo demonstrated a significantly longer blood half‐life (*t*_1/2_ = 7.42 ± 0.83 h) compared to free IR806 (*t*_1/2_ = 2.97 ± 0.32 h). ictLipo accumulated gradually at the tumor site, with peak enrichment observed at 8 h (Figure S19C), and showed a gradual decline in fluorescence until 24 h, indicating prolonged retention at the tumor site. *Ex vivo* imaging results corroborate this trend, revealing higher fluorescence intensity in tumors compared to major organs at both 8 and 24 h post‐injection (Figures S19D,E). This suggests superior tumor‐targeting capability of ictLipo. Additionally, ictLipo exhibited increased accumulation in the liver and kidney, likely due to the enhanced uptake of positively charged liposomes by the reticuloendothelial system. Consequently, the plasma clearance (CL) of ictLipo was 1.56‐fold lower than that of free IR806 (Figure S19B). In summary, ictLipo demonstrates effective circulation in the bloodstream and substantial accumulation in secondary organs, with favorable blood clearance and minimal toxicity under the experimental conditions, as confirmed by subsequent biosafety assessments in mice (Figure S24).

**FIGURE 5:**
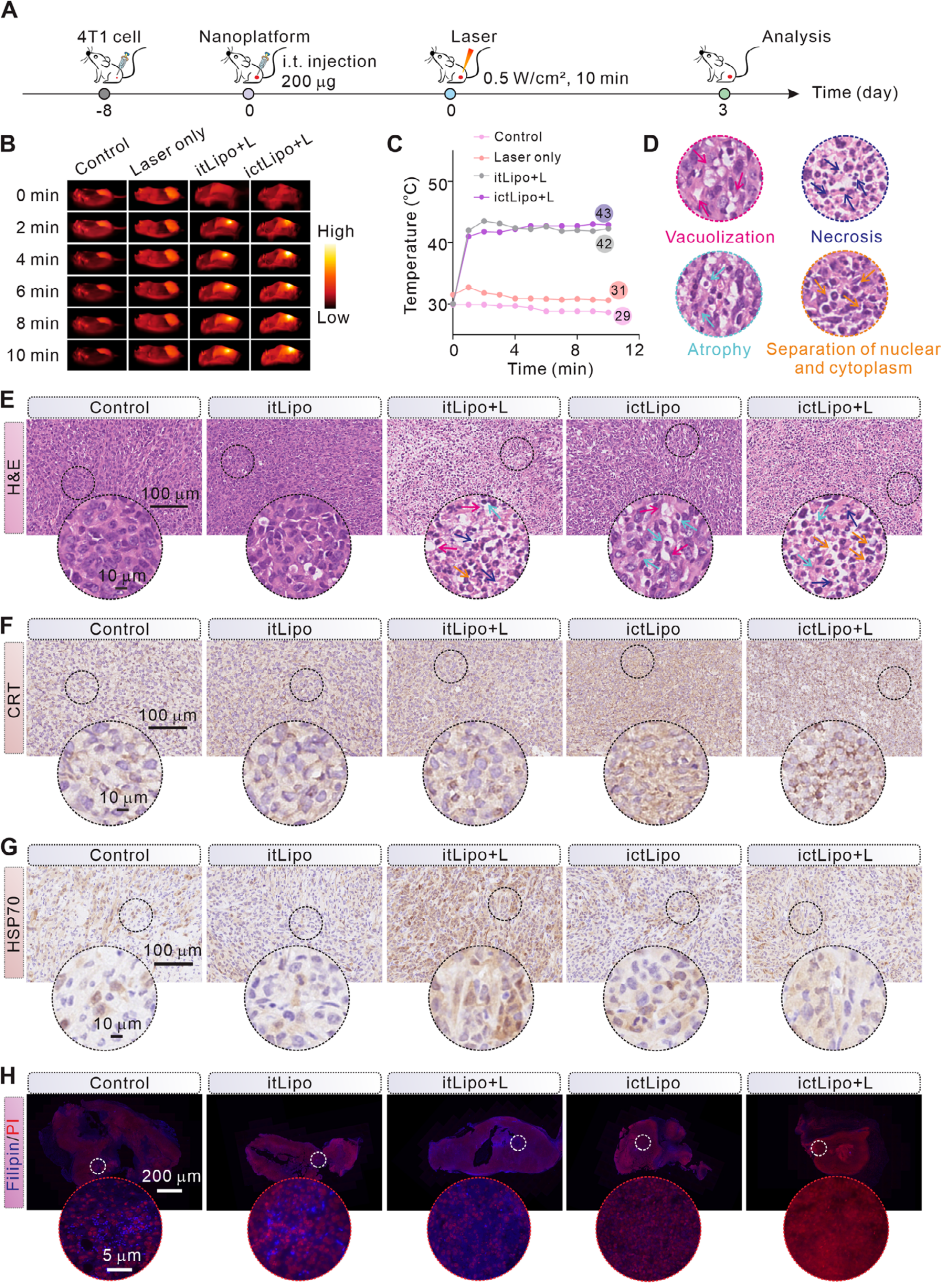
In vivo antineoplastic mechanisms of ictLipo. (A) Treatment processes of nanoplatforms in 4T1 tumor‐bearing mice. (B,C) Thermal images (B) and corresponding temperature curves (C) of tumor sites after injecting PBS or itLipo or ictLipo followed with/without 808 nm laser treatments (0.5 W cm^−2^). (D,E) H&E staining of tumor slices of mice with the indicated treatments. The arrows of red, blue, green, and orange colors represent vacuolization, necrosis, atrophy, and separation of nuclear and cytoplasmic, respectively. The black dotted circles indicate the enlarged areas. Scale bars are 100 µm. (F,G) Immunohistochemical staining of CRT (F) and HSP70 (G) of tumor slices from mice after indicated treatments. Scale bars are 100 µm. (H) Immunofluorescent staining of Filipin and PI of tumor slices from mice after the indicated treatments. Cholesterol was stained with Filipin, and the cell nucleus was stained with PI, respectively. Scale bars are 200 µm.

The CD‐CT/PTT performance by ictLipo in vivo was studied on the TNBC mouse tumor model (Figure 5A). In a typical experiment, 4T1 tumor‐bearing mice were intratumorally injected with assigned probes followed by laser illumination at 2 h, and the temperature of tumor sites was recorded by an infrared camera. As shown in Figures 5B,C, in comparison to the control and laser only group, the temperature of tumor sites in groups itLipo and ictLipo increased rapidly under 800 nm laser irradiation. After treatments for 3 days, mice were euthanized, and tumors were collected.

According to histological H&E (hematoxylin and eosin) staining of the tumor slices (Figures 5D,E), we found that ictLipo + L induced severe tumor damage, including vacuolization, necrosis, atrophy, and separation of nuclear and cytoplasm. CRT immunofluorescent staining of tumor slices showed that high CRT expression was detected in the ictLipo + L group, verifying its role on in vivo ICD induction (Figure 5F). Immunohistochemical staining of HSP70 revealed that itLipo + L induced high expression of HSP70 while ictLipo + L exhibited less HSP70 expression, confirming the successful PTT implementation of the ictLipo + L (Figure 5G). Besides, on the basis of Filipin fluorescent staining of tumor slices (Figure 5H), we found that group ictLipo exhibited dim blue fluorescence signals compared to the groups of control, itLipo, and itLipo + L, indicating that ChOx in ictLipo can deplete cholesterol in tumors. Significantly, group ictLipo + L showed a minimal Filipin intensity, demonstrating that the ictLipo is able to deplete cholesterol in the tumor site under laser irradiation and verifying its cholesterol depletion in TME.

### 2.6 In Vivo Immune Response and T Cell Exhaustion Relief

**FIGURE 6:**
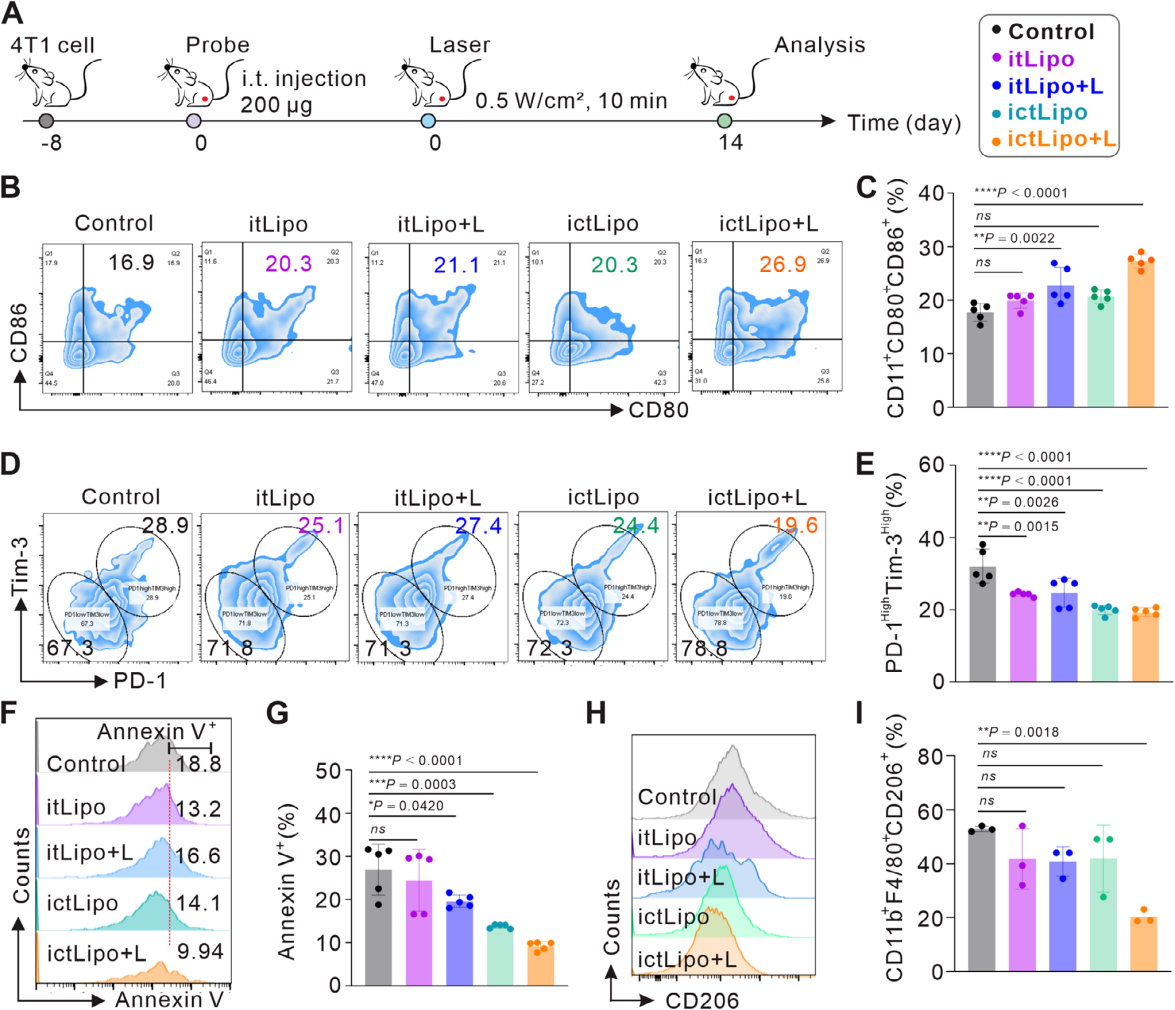
ictLipo induces robust in vivo antitumor immune responses. (A) Treatment processes of nanoplatforms in 4T1 tumor‐bearing mice. (B,C) The efficacy of DC maturation (CD11^+^CD80^+^CD86^+^) in lymph glands by a FCM analysis (B) and corresponding statistical results (C), *n* = 5. (D,E) The FCM analysis of exhaustion (CD3^+^CD8^+^PD‐1^+^Tim‐3^+^) (D) of tumor‐infiltrating CD8^+^ T cells and corresponding statistical results (E). (F,G) The FCM analysis of apoptosis (CD3^+^CD8^+^Annexin V^+^) (F) of tumor‐infiltrating CD8^+^ T cells and corresponding statistical results (G). (H,I) The percentage of macrophages by a FCM analysis (H) and statistical results (I). *n* = 3. *p < 0.05, **p < 0.01, ****p < 0.0001, and ns: not significant (*p* > 0.05), analyzed by one‐way ANOVA, followed by Dunnett's multiple comparisons test. Data represent mean ± s.d.

Subsequently, we evaluated the in vivo antitumor immune responses by ictLipo in the 4T1 tumor‐bearing mouse model (Figure 6A). After treatment with assigned schemes for 14 days, the mice were euthanized, and lymph glands and tumors were collected. According to an analysis of single‐cell suspension of the lymph glands (Figures 6B,C), we found that ictLipo + L triggered more DC maturation (26.9%) than other groups. The luxuriant cholesterol in the TME was recently reported to exhaust CD8^+^ T cells through upregulating immune checkpoints such as PD‐1 and TIM‐3 [34, 35]. Inspired by the excellent cholesterol depletion ability of ictLipo + L to the TME (Figure 5H), we further evaluate the influence of ictLipo + L on CD8^+^ T cell exhaustion. As shown in Figures 6D,E, ictLipo + L treatment showed less proportion of PD‐1^high^Tim‐3^high^ T cells (about 19.6%) compared with other groups (gated on CD3^+^CD8^+^, Figure S20). Besides, the cell viability of CD8^+^ T cells was studied by Annexin V staining and FCM analysis (Figures 6F,G). The results indicated CD8^+^ T cells with ictLipo + L treatment showed less cell apoptosis, about 9.94%. Furthermore, the macrophages polarized from M2 (gated on CD11b^+^F4/80^+^CD206^+^, Figure S21) to M1 (gated on CD11b^+^F4/80^+^CD206^−^, Figure S21), which further implies the powerful immune responses evoked by ictLipo + L (Figures 6H,I).

### 2.7 Antimetastasis Effect of ictLipo Plus αPD‐1 Antibody

**FIGURE 7:**
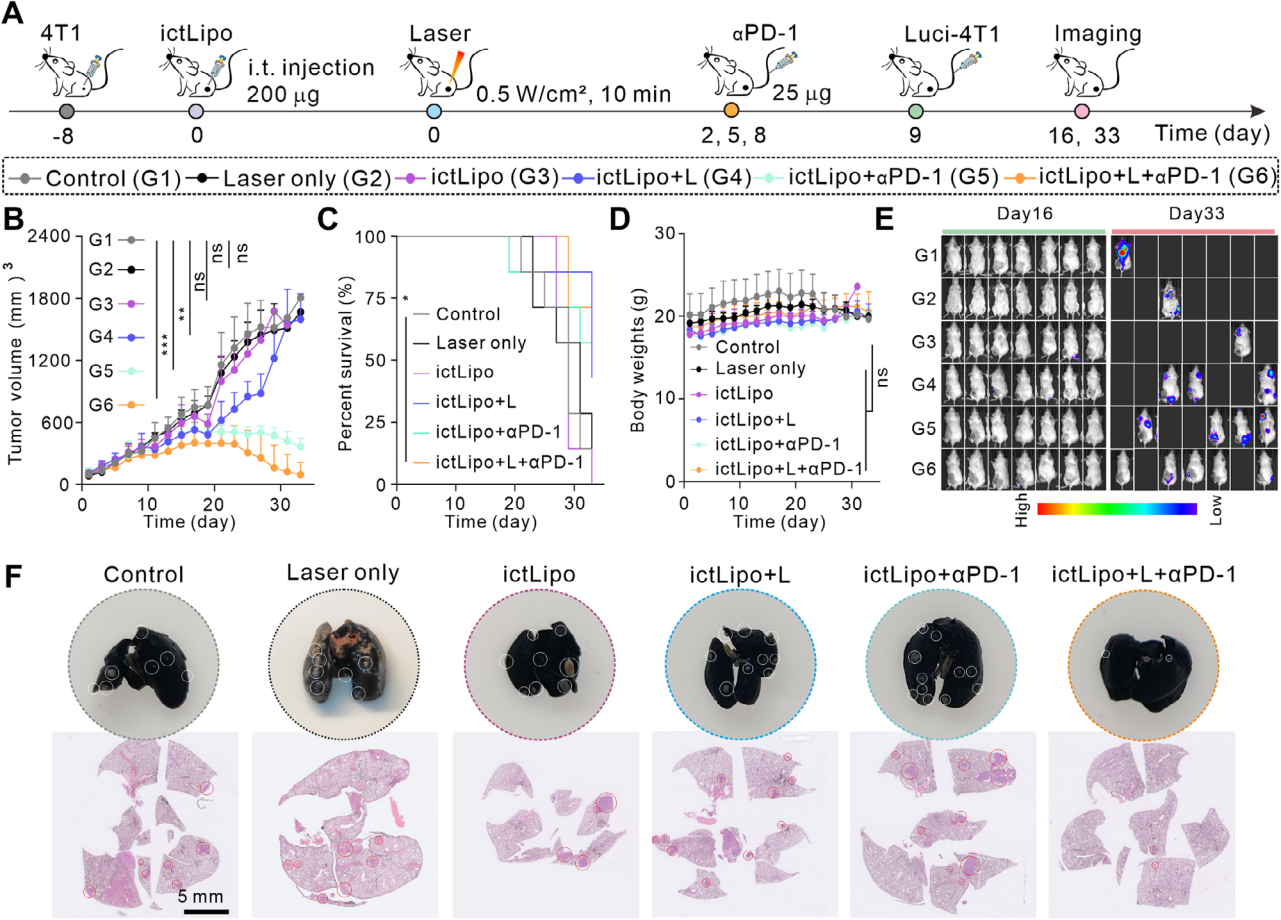
ictLipo combined with αPD‐1 for tumor metastasis inhibition in the 4T1 tumor model. (A) Treatment process. (B) Tumor volume curves of mice after the indicated treatments (*n* = 7). (C,D) The survival percentage (C) and body weights (D) of mice. (E) The luciferin bioluminescence images of mice on day 16 and day 33. (F) Representative images of lung tissues that were stained with India ink (upper) and H&E (lower). Scale bars are 5 mm. *p < 0.05, **p < 0.01, ***p < 0.001, and ns: not significant (*p* > 0.05), analyzed by one‐way ANOVA, followed by Dunnett's multiple comparisons test. Data represent mean ± s.d.

The robust immune responses induced by ictLipo + L motivate us to explore the immunotherapy of ictLipo combined with PD‐1 checkpoint blockade. The treatment process is illustrated in Figure 7A. The 4T1 tumor‐bearing mice received probe injection and laser illumination on day 0 followed by αPD‐1 antibody injection on days 2, 5, and 8. On day 9, mice were *i.v*. injected with luci‐4T1 cells and their luciferin bioluminescence was recorded by the IVIS system on days 16 and 33. Body weights and tumor sizes of mice were measured every two days after treatments. As shown in Figure 7B, tumors in group control (PBS injection, G1) and group laser only (only laser treatment, G2) grew rapidly. ictLipo + L (with laser treatment, G4) and ictLipo (without laser treatment, G3) slowed the growth of tumors in the first 18 days, but then the tumors still grew rapidly. By comparison, group ictLipo + αPD‐1 (PD‐1 antibody, G5) obviously inhibited the subcutaneous tumors. Specifically, ictLipo + L + αPD‐1 (with laser treatment, G6) had a better tumor inhibition effect compared to the ictLipo + αPD‐1, suggesting that CD‐CT/PTT realized by the ictLipo + L can be synergistic with PD‐1 checkpoint blockade for tumor inhibition. We further evaluated the survival rate of mice after different treatments. As shown in Figure 7C, ictLipo + L + αPD‐1‐treated mice had a 71.49% survival rate, which was higher than any of the other groups. Meanwhile, the body weights of mice during treatments had no obvious changes (Figure 7D). On the basis of bioluminescence imaging (Figure 7E), we found that mice with ictLipo + L + αPD‐1 displayed significantly lower fluorescence signals than other groups. Furthermore, India ink staining (Figure 7F, upper) and H&E staining (Figure 7F, lower) demonstrated fewer metastasis niduses in lung tissues in group ictLipo + L + αPD‐1 than others. All considered, ictLipo + L implemented CD‐CT/PTT combination with αPD‐1 can inhibit tumor growth and metastasis and prolong mouse survival in the TNBC mouse carcinoma model.

### 2.8 Immune Mechanism of ictLipo Plus PD‐1 Antibody

**FIGURE 8:**
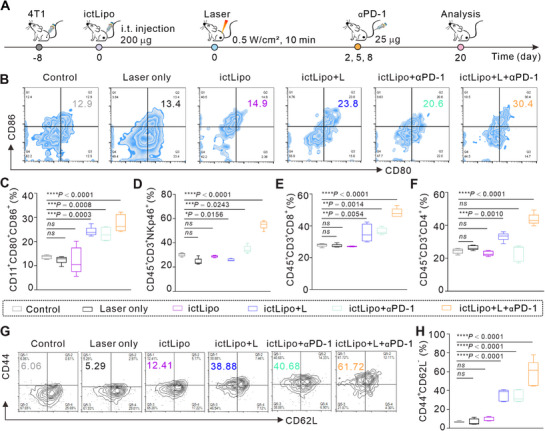
Immunological response after ictLipo combined with αPD‐1. (A) Treatment process (*n* = 5). (B,C) Representative flow cytometer images (B) and statistical results (C) of DC maturation in the lymph gland. (D–F) Statistical results of NK cells (D), CD8^+^ T cells (E), and CD4^+^ T cells (F) of tumors from mice received various treatments. (G) Representative flow cytometer images (G) and statistical results (H) of memory T cells in tumors. *p < 0.05, **p < 0.01, ***p < 0.001, ****p < 0.0001, and ns: not significant (*p* > 0.05), analyzed by one‐way ANOVA, followed by Dunnett's multiple comparisons test. Data represent mean ± s.d.

Next, we explored the antitumor immune mechanisms of CD‐CT/PTT combined with αPD‐1. The treatment process is illustrated in Figure 8A. After treatment with the indicated schemes for 20 days, mice were sacrificed, and their tumors and major immune organs (lymph glands and tumors) were collected. As can be seen from Figures 8B,C, both laser irradiation and αPD‐1 enhanced the DC maturation of ictLipo. Specifically, group ictLipo + L + αPD‐1 stimulated about 30% DC maturation in the tumor‐infiltrating lymph glands, which is obviously higher than other groups. The excellent ability to promote DC maturation of ictLipo + L + αPD‐1 will guarantee the following antigen‐presenting and T‐cell priming. Subsequently, we examined the natural killer (NK) cells (gated on CD45^+^CD3^+^NKp46^+^, Figure S22), tumor‐infiltrated cytotoxic T cells (gated on CD45^+^CD3^+^CD8^+^), and helper T cells (gated on CD45^+^CD3^+^CD4^+^). As shown in Figure 8D, ictLipo + L + αPD‐1 treatment increased the NK cells in tumors. In addition, there was a significant increase in both cytotoxic T cells (Figure 8E) and helper T cells (Figure 8F) in ictLipo + L + αPD‐1‐treated tumors. Furthermore, the memory T cells (gated on CD4^+^CD8^+^CD44^+^CD62L^−^, Figure S23) were investigated in tumors after different treatments. As can be seen from Figures 8G,H, we found that ictLipo could augment memory T cells, and its effects were enhanced upon combining with laser illumination or αPD‐1 administration. Strikingly, ictLipo + L + αPD‐1 significantly elevated the T memory cells. All considered, these data suggest that ictLipo‐mediated CD‐CT/PTT can boost robust antitumor innate and acquired immune responses, which results in excellent tumor therapeutic efficacy and metastasis inhibition capacity.

**FIGURE 9:**
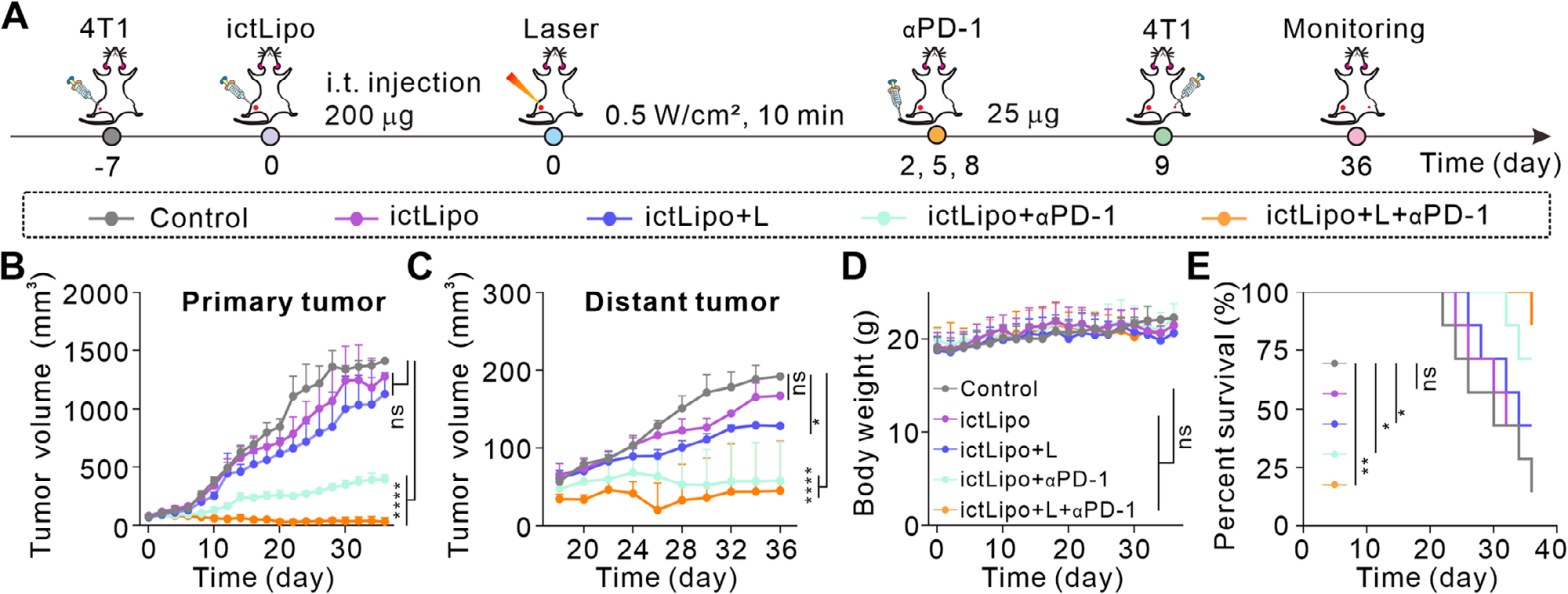
ictLipo combined with αPD‐1 for distant tumor inhibition in a bilateral 4T1 tumor model. (A) Schematic illustration of tumor treatment processes. (B,C) Growth curves of primary (B) and distant (C) tumors. (D) Body weights of mice after various treatments. (E) Survival rate of tumor‐bearing mice with different treatments.

Moreover, we utilized a bilateral TNBC mouse model to further test the distant tumor inhibition ability of ictLipo (Figure 9A). In a typical experiment, one hind leg of the BALB/c mouse was injected with 4T1 cells. Seven days later, mice were treated with designated schemes. Then, mice in groups ictLipo + αPD‐1 and ictLipo + L + αPD‐1 were *i.v*. injected with αPD‐1 antibody on days 2, 5, and 8. On day 9, all mice were inoculated subcutaneously with 4T1 cells at another hind leg flank as the distant tumor. As shown in Figures 9B,C, ictLipo + L + αPD‐1 not only inhibited the growth of primary tumors but also suppressed the distant tumors. The body weights of mice in different groups exhibited no obvious changes (Figure 9D). Unsurprisingly, ictLipo + L + αPD‐1‐treated mice had the highest survival rate of 85.71% compared to the other four groups (Figure 9E). Keeping all these exciting results in mind, we may conclude that our designed ictLipo nanoplatform can induce robust antitumor immune responses by combination with local laser irradiation and αPD‐1 checkpoint blockade, giving rise to excellent distant tumor suppression.

Finally, the biocompatibility of ictLipo was thoroughly assessed both in vitro and in vivo. In vitro evaluations included hemolysis tests and cytotoxicity assays on normal cells. As depicted in Figure S24A, ictLipo demonstrated negligible hemolytic activity, with a hemolysis ratio of less than 5% even at a high concentration of 600 µg mL^−1^. Additionally, ictLipo exhibited no cytotoxicity towards normal cells, including MLE‐12 (murine lung epithelial) and BEAS‐2B (human bronchial epithelial) cells, even at a concentration of 200 µg mL^−1^ (Figure S24B). In vivo, a comprehensive long term toxicity study was conducted. Data indicated that ictLipo treatment did not produce significant alterations in organ body weights (Figure S24C), hematological parameters, or physicochemical serum indicators compared to the control group (Figure S24D). Histological analysis of major organs (heart, liver, kidney, lung, spleen, and intestine) revealed no apparent cell damage or pathological abnormalities following treatment (Figure S24E).

## 3 Conclusions

In summary, we have successfully constructed a cholesterol‐depleted nanoliposome (ictLipo) that can ameliorate CT/PTT and stimulate robust antitumor immune responses. The ictLipo can initiate hypoxia‐activated CT and strengthen PTT via cholesterol depletion (CD), thus inducing strong ICD responses. Importantly, the cholesterol depletion ability of ictLipo can also reverse CD8^+^ T cell exhaustion, thus achieving favorable immunotherapy by combination with αPD‐1 checkpoint blockade. Our work demonstrated that tumor metastasis and invasion and T cells exhaustion can be remitted by simply depleting cholesterol, providing a facile and simple strategy for TNBC therapy.

## 4 Materials and Methods

### 4.1 Materials

IR806, cholesterol oxidase (ChOx), tirapazamine (TPZ), [(6Z,9Z,28Z,31Z)‐heptatriaconta‐6,9,28,31‐tetraen‐19‐yl] 4‐(dimethylamino) butanoate (DLin‐MC_3_‐DMA), 1,2‐dimyristoyl‐sn‐glycero‐3‐methoxypolyethylene glycol 2000 (Sunbright DMG‐PEG_2000_), 1,2‐distearoyl‐sn‐glycero‐3‐phosphocholine (18: 0 PC, DSPC), and cholesterol were bought from Shanghai Aladdin Biochemical Technology Co., LTD. ATP assay kit, and 1% TMB solution were purchased from Solarbio Science & Technology Co., Ltd. The cell counting kit (CCK‐8) was obtained from DOJINDO. All antibodies used in flow cytometry were purchased from BioLegend. All the reagents used to culture cells (DMEM, RPMI‐1640, PBS, FBS, and penicillin‐streptomycin) were obtained from Gibco.

### 4.2 Synthesis of ictLipo

The method used to synthesize Lipo is according to a previously reported article [25]. In brief, a lipid solution (the molar ratios of DLin‐MC_3_‐DMA:DSPE:DMG‐PEG_2000_:ChOl = 50: 10: 1.5: 38.5) was passed through an Avanti liposome extruder (800, 400, and 200 nm) for 30 times at 4°C to get liposome nanoparticles. For ictLipo synthesis, IR806, ChOx, and/or TPZ (mass ratio _(IR806: Lipo)_ = 1: 5; mass ratio _(ChOx:Lipo)_ = 1: 2; mass ratio _(TPZ:Lipo)_ = 1: 40) were dissolved in lipid solution, followed by passing through lipid extruder. Then the solution was centrifuged two times to obtain ictLipo. The loading efficacy of IR806 and TPZ, was calculated by the standard curve method of UV–vis spectra. The loading efficacy of ChOx was measured by a BCA protein assay and a nanodrop measurement. The protein content of cholesterol oxidase (ChOx) in the remaining supernatant and the total ictLipo was quantified using the BCA protein assay and nanodrop methods. The absorbance at 280 nm was recorded, corresponding to the ChOx concentration. The encapsulation efficiency of ChOx in the total ictLipo was then calculated using the formula: (ChOx content in total ictLipo − ChOx content in supernatant) / (ChOx content in total ictLipo) × 100%.

### 4.3 Photothermal Property of ictLipo

The photothermal performance of 100 µg mL^−1^ iLipo, ictLipo, and an equivalent concentration of IR806 solution was recorded under an 808 nm laser irradiation for 5 min at 0.5 W cm^−2^ by a thermal detector (CENTER, 306). Ultrapure water was used as a control.

For photothermal conversion efficiency (*η*) calculation, 100 µg mL^−1^ Lipo, ictLipo, and an equivalent concentration of IR806 were exposed to 808 nm laser irradiation for 5 min at 0.5 W cm^−2^ and laser off for 10 min. The temperature value was recorded, and the photothermal conversion efficiency was calculated by the following equation:

$$\eta =hS\left(T_{\max}-T_{\mathrm{sur}}\right)-Q_{S}/I\left(1-10^{-A_{808}}\right)$$

where *η* represents the photothermal conversion efficiency, *h* represents heat transfer coefficient, *S* represents the basal area of the detachable 96‐well plate, *T*_max_ represents the highest temperature reached of ictLipo exposed to laser illumination, *T*_sur_ represents the ambient temperature, and *Q*_s_ represents heat dissipated from the light absorbed by the container and water. *I* represents the power of the laser used in the experiment, *A*_808_ represents the absorption intensity at 808 nm of ictLipo solution in UV–vis spectra.

For photothermal stability evaluation, 100 µg mL^−1^ ictLipo and an equivalent concentration of IR806 were exposed to repetitive laser on/off cycles at 0.5 W cm^−2^.

### 4.4 Cholesterol Oxidase Activity Evaluation

The cholesterol oxidase activity was measured by a colorimetric method [29]. In the presence of oxygen, cholesterol oxidase could catalyze cholesterol to generate 4‐cholesten‐3‐one and H_2_O_2_. The 4‐amino‐antipyrine could react with phenol in the presence of H_2_O_2_ and peroxidase, followed by red substance generation. Therefore, the absorption intensity at 500 nm could evaluate the enzyme activity of cholesterol oxidase. Briefly, the assay solution consisted of 1 mM 4‐amino‐antipyrine, 5 mM phenol, 5 U/mL peroxidase, and sodium phosphate buffer (20 mM, pH = 7.0). 300 µg cholesterol dissolved in dimethylformamide containing 5% TritonX‐100 was added to 1.0 mL assay solution and preincubated for 3 min. Then 20 µg mL^−1^ ChOx was added and incubated at different temperatures (4°C, 25°C, 37°C, and 45°C) for another 5 min. After that, the mixture was boiled in a water bath for 2 min and placed in an ice bath for 2 min. Finally, the 500 nm absorption of the mixture was read in a MultiScan reader.

### 4.5 Cell Culture

Mouse triple‐negative breast cancer cells 4T1 were procured from ATCC. Mouse bone marrow‐derived dendritic cells DC2.4 and mouse T lymphocytes cells CTLL2, and the cell culture media for DC2.4 and CTLL2 cells were obtained from Procell Life Science & Technology Co. Ltd. Luci‐4T1 was obtained by lentiviral transfection. 4T1 cells and luci‐4T1 cells were cultured in DMEM supplemented with 10% FBS and 1% penicillin‐streptomycin antibiotics. DC2.4 cells were cultured in RPMI‐1640 supplemented with 10% FBS and 1% penicillin‐streptomycin antibiotics. CTLL2 cells were cultured in RPMI‐1640 supplemented with 10% FBS, 100 U/mL IL‐2, 1 µg mL^−1^ Con A, and 1% penicillin‐streptomycin antibiotics. All cells were cultured in a humidified incubator with 5% CO_2_ at 37°C. All cells tested negative for mycoplasma contamination and rodent pathogens.

### 4.6 Cellular Uptake of ictLipo

4T1 cells (5 × 10^5^) were seeded in 12‐well plates. Then the cell samples were incubated with 100 µg mL^−1^ FITC‐conjugated ictLipo for different time intervals. The cellular uptake situation was evaluated by fluorescent intensity via flow cytometry detection (FCM, Agilent, Novoexpress).

### 4.7 Intracellular Cholesterol Imaging

4T1 cells (2×10^5^) were seeded in confocal dishes. Then the cell samples were incubated with 100 µg mL^−1^ itLiop, ictLipo, and an equivalent concentration of ChOx for 12 h followed by 808 nm laser illumination at 0.5 W cm^−2^ for 5 min or not. Afterward, cell samples were stained with Filipin (50 µg mL^−1^, 2 h) and imaged by Confocal Laser Scanning Microscopy (Sunny Optical Technology, SOPTOP CLSM610, China).

### 4.8 Wound Healing Assay

4T1 cells (5 × 10^5^) were seeded in 12‐well plates. And then a sterile plastic micropipette tip was used to stimulate an in vivo wound. Subsequently, the cell samples were incubated with 100 µg mL^−1^ itLiop, ictLipo, and an equivalent concentration of ChOx for 12 h followed by 808 nm laser illumination at 0.5 W cm^−2^ for 5 min or not. Finally, the wound healing process of cell samples was imaged in an optical microscope (Olympus, CKX53).

### 4.9 Western Blot Assay

4T1 cells were seeded in 6‐well plates overnight. Then the cell samples were incubated with 100 µg mL^−1^ ictLiop, ictLipo + L (with 808 nm laser irradiation, 0.5 W cm^−2^, 5 min), and an equivalent concentration of ChOx for 12 h. Thereafter, cell samples were homogenized in 40 µL RIPA buffer (Beyotime) containing protease inhibitors, and centrifuged at 13,000 rpm for 10 min at 4°C to concentrate proteins. After that, proteins were extracted from cell samples and quantified with a BCA kit. Proteins were fractionated by SDS‐PAGE and transferred to 0.22 µm PVDF membranes (Millipore, Darmstadt, Germany). Then, the membrane was incubated with anti‐HSP70 (1: 1000) followed by secondary antibody (1: 200) incubation. At last, the protein bands were acquired by a gel imaging system (Odyssey, C1x) and the according gray values were quantified by Image J software.

### 4.10 In Vitro Therapeutic Efficacy of ictLipo Evaluation

4T1 cells were seeded in 6‐well plates. Then, cells were incubated with different concentrations of iLipo, itLipo, icLipo, and ictLipo (0, 50, 100, 150, and 200 µg mL^−1^) for 12 h followed by laser irradiation or not, and another (808 nm laser, at 0.5 W cm^−2^ for 5 min). After another 4 h of incubation, the cell viability was measured by a CCK8 kit.

### 4.11 In Vitro ICD Evaluation

4T1 cells (2×10^5^) were seeded in confocal dishes overnight. Then cells were treated with (1) control, (2) itLipo, (3) itLipo + L, (4) ictLipo, (5) ictLipo + L (100 µg mL^−1^, 12 h, + L represents 808 nm laser illumination at 0.5 W cm^−2^ for 5 min). Afterward, the supernatant was collected for HMGB1 and APT measurements while cell samples were washed, fixed, permeabilized, and stained with Calreticulin‐ER antibody at 37°C for 30 min. Thereafter, a secondary antibody conjugated with FITC was added and incubated for another 1 h. Cell samples were imaged via CLSM. The same procedures to prepare cell samples and fluorescent intensity were measured by a FCM analysis.

### 4.12 In Vitro DC Maturation Evaluation

4T1 cells were seeded in 6‐well plates and received various treatments. After that, the supernatant of cell samples was collected and mixed with DC2.4 culture media to treat DC2.4 for 12 h. Then, DC2.4 cells were collected and stained with antibodies for flow cytometer analysis to evaluate the DC maturation (gated on CD11c^+^CD80^+^CD86^+^). For BMDCs, a standard protocol was utilized to separate and culture BMDCs [36]. First, Bone marrow cells were harvested from tibiae and femurs of BALB/c mice. Then the collected bone marrow cells were suspended in 1640‐RPMI media with 10% FBS, 1% penicillin/streptomycin, 20 ng mL^−1^ GM‐CSF, and 10 ng mL^−1^ IL‐4. After 6 days of cultivation, the BMDCs were obtained and further received various treatments. At last, the maturation of BMDCs was examined by an FCM analysis (BD Bioscience, FACSymphony A5). All antibodies used in immune response experiments are listed in Table S1.

### 4.13 Pharmacokinetic Study and In Vivo Fluorescence Imaging and Biodistribution

The 4T1 tumor‐bearing mice were intravenously injected with free IR806 (100 µg) and ictLipo (containing 100 µg IR806) for in vivo tracking under an IVIS imaging system from RWD Life science (MOIS HT Small Animal In Vivo Optical Imaging System, China). At designated time points, 200 µL blood samples were collected, centrifuged at 5000 rpm for 10 min to separate plasma, and then processed by methanol deproteinization followed by centrifugation at 10000 rpm for 10 min. IR806 fluorescence in the supernatant was measured, and a standard curve was created to determine IR806 concentration in the samples. Mice were imaged at scheduled intervals, and at 8 and 24 h post‐injection, major organs and tumors were harvested for ex vivo fluorescence imaging.

### 4.14 In Vivo ICD Measurements and Cholesterol Regulation

Female BALB/c mice (four weeks) were purchased from Beijing Hua Fu Kang Biotechnology Co. Ltd., and all the mice experiments were implemented in accordance with protocols approved by the Animal Experimental Ethics Committee of Fujian Normal University (Approval No. IACUC‐20220006). 4T1 tumor‐bearing mice received various nanoplatform treatments (200 µg itLipo and ictLipo per mouse) via intratumor injection and exposed to laser illumination (808 nm, 0.5 W cm^−2^, 10 min) or not after 1  h injection. An infrared camera was utilized to record the temperature changes in the tumor area of mice. Three days later, the tumors were collected and sliced for H&E histological staining, CRT and HSP70 immunohistochemical staining, and Filipin/PI staining.

### 4.15 Evaluation of In Vivo DC Maturation and T Cell Exhaustion

4T1 tumor‐bearing mice received various treatments with assigned schemes (*n* = 5). After 14 days, mice were sacrificed and the lymph gland and tumor were collected and digested to single‐cell suspension. The cellular suspension from the lymph gland was used for DC maturation analysis (gated on CD11c^+^CD80^+^CD86^+^). T cells within the tumors were analyzed for Tim3, PD‐1, and Annexin V content. The polarization of macrophages within tumors was also analyzed (gated on CD11b^+^F4/80^+^CD206^+^ and CD11b^+^F4/80^+^CD206^−^).

### 4.16 In Vivo TNBC Therapy by ictLipo

4T1 tumor‐bearing mice received various treatments (*n* = 7) and αPD‐1 antibody injections on 2, 5, and 8 days with assigned schemes. Tumor volume and body weights were recorded every two days. Nine days later, mice were intravenously injected with luci‐4T1 cells (1 × 10^6^ per mouse) and the bioluminescence of mice was recorded by an IVIS on day 16 and day 33. On day 33, one mice of each group were sacrificed, lung tissues were collected. Afterward, lung tissues were conducted for India ink staining and H&E staining to further analyze the metastatic nodes. For the distant tumor inhibition model, 4T1 tumor‐bearing mice received various treatments (*n* = 7) and αPD‐1 antibody injections on 2, 5, and 8 days with assigned schemes. Nine days later, 4T1 cells were subcutaneous injected in the other side of BALB/c mice. The volume of the primary tumor and distant tumors and body weights were recorded every two days.

### 4.17 Immune Mechanism of In Vivo Tumor Therapy by ictLipo

4T1 tumor‐bearing mice were received various treatments (*n* = 5) as assigned schemes. Mice were sacrificed on day 20, and lymph glands and tumors were collected for flow cytometer analysis. DC maturation of DC in lymph glands was gated on CD11c^+^CD80^+^CD86^+^. NK cells in tumors were gated on CD45^+^CD3^+^NKp46^+^. T cells in tumors were gated on CD45^+^CD3^+^, while memory T cells were gated on CD4^+^CD8^+^CD44^+^CD62L^−^.

### 4.18 Hemolysis Study

The hemocompatibility of Lipo and ictLipo was assessed using a hemolysis test. Briefly, mouse blood was collected, and erythrocytes were isolated by centrifugation at 3000 rpm for 15 min. The erythrocytes were washed with PBS and diluted to 2% concentration. Then, these cells were incubated with various concentrations of Lipo and ictLipo for 1 h. The hemolysis rates were determined by measuring the absorbances of the mixtures at 576 nm.

### 4.19 Long‐Term Toxicity Assay

Twelve healthy BALB/c mice were divided into two groups (*n* = 6 per group) as follows: PBS and ictLipo. After a long‐term treatment, the mice were sacrificed, and major organs (heart, liver, spleen, lung, kidney, and intestine) were collected for H&E staining, and their weights were recorded. Besides, blood samples from the treated mice were analyzed for blood routine and physicochemical serum analysis.

### 4.20 Statistical Analysis

Statistical analysis was performed by GraphPad Prism (9.0) and evaluated by analysis of variance (ANOVA). The significance of the data is determined by the Student's test: *p < 0.05, **p < 0.01, ***p < 0.001, ****p < 0.0001, and ns: not significant (*p* > 0.05).

## Conflicts of Interest

The authors declare no conflicts of interest.

## Supporting information




**Supporting File 1**: exp270097‐sup‐0001‐SuppMat.docx

## Data Availability

The data that supports the findings of this study are available in the supplementary material of this article.
