# Supplementary material for: Cholesterol Metabolism Regulated Nanoliposome Ameliorates Chemo/Photothermal Therapy Reversing CD8+ T Cell Exhaustion
Source: Exploration (Beijing). 2025 Dec 7;6(1):20240123. doi: 10.1002/EXP.20240123 (PMC12970277; doi:10.1002/EXP.20240123)
Supplement: Supplementary file 1 — Supporting File 1: exp270097‐sup‐0001‐SuppMat.docx [file EXP2-6-20240123-s001.docx]

Supporting Information

Cholesterol Metabolism Regulated Nanoliposome Ameliorates Chemo/Photothermal Therapy Reversing CD8^+^ T Cell Exhaustion

*Panpan Xue ^a,b^, Tingjie Bai ^b^, Huilan Zhuang ^b^,* *Angelo H. All ^c^, Shuangqian Yan ^b,^*, Xuemei Zeng ^a,^**

^a^ Key Laboratory of Microbial Pathogenesis and Interventions of Fujian Province University, Biomedical Research Center of South China, College of Life Sciences, Fujian Normal University, 1 Keji Road, Fuzhou 350117, PR China

^b^ Strait Institute of Flexible Electronics (SIFE, Future Technologies), Fujian Normal University and Strait Laboratory of Flexible Electronics (SLoFE), Fuzhou, China

^c^ Sir Run Run Shaw Building, Office RRS844, Ho Sin Hang Campus, Hong Kong Baptist University, Hong Kong SAR, China

* Address correspondence to: Shuangqian Yan; [ifeshqyan@fjnu.edu.cn](mailto:ifeshqyan@fjnu.edu.cn) and Xuemei Zeng; [xmzeng@fjnu.edu.cn](mailto:xmzeng@fjnu.edu.cn)

**KEYWORDS:** Cholesterol metabolism, checkpoint blockade, photothermal therapy, T cell exhaustion

| Antibodies | Source | Catalogue |
| --- | --- | --- |
| FITC anti-mouse/human CD11c | Biolegend | Cat#117305 |
| PE anti-mouse/human CD80 | Biolegend | Cat#104707 |
| APC anti-mouse CD86 | Biolegend | Cat#105011 |
| PE/Cyanine7 anti-mouse CD3 | Biolegend | Cat#100219 |
| APC/Cyanine7 anti-mouse CD8α | Biolegend | Cat#100714 |
| PE anti-mouse CD279 (PD-1) | Biolegend | Cat#114117 |
| PE/Dazzle 594 anti-mouse Ki67 | Biolegend | Cat#652428 |
| APC anti-mouse CD366 (Tim-3) | Biolegend | Cat#119706 |
| FITC Annexin V | Biolegend | Cat#640906 |
| PE anti-mouse CD45 | Biolegend | Cat#103105 |
| APC anti-mouse CD4 | BD Pharmingen | Cat#561091 |
| PerCP-Cy5.5 anti-mouse CD8α | BD Pharmingen | Cat#551162 |
| FITC anti-mouse CD335(NKp46) | Biolegend | Cat#137605 |
| PE/Cyanine5 anti-mouse CD45 | Biolegend | Cat#103110 |
| APC/Cyanine7 CD11b | Biolegend | Cat#101226 |
| PE/CF594 anti-mouse F4/80 | BD Pharmingen | Cat#565613 |
| Alexa Flour647 anti-mouse CD206 | BD Pharmingen | Cat#565250 |
| BV605 anti-mouse CD11c | BD Pharmingen | Cat#744179 |
| FITC anti-mouse CD4 | Biolegend | Cat#100406 |
| APC anti-mouse CD44 | Biolegend | Cat#103012 |
| BV650 anti-mouse CD62L | BD Pharmingen | Cat#564108 |

**Table S1.** **A list of antibodies used in flow cytometric experiments.**


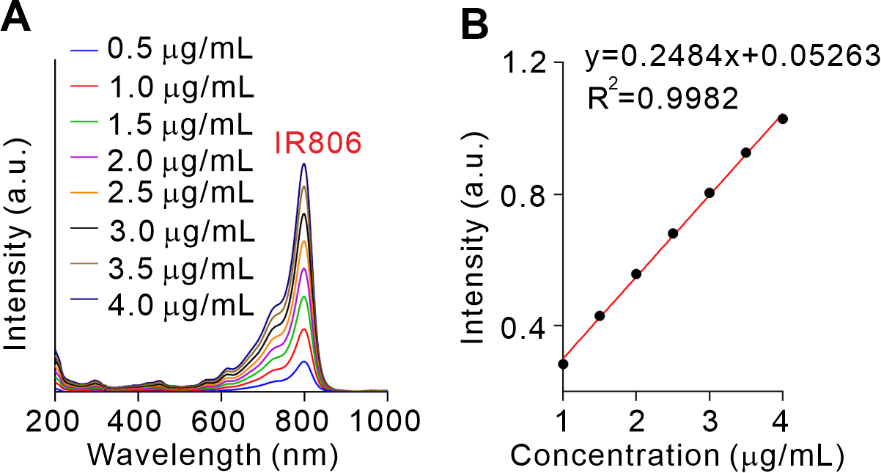


**Fig. S1**. (**A**) UV-visible spectra of various concentrations of IR806 solution. (**B**) Linear relation between concentration and corresponding intensity at 800 nm from (**A**).

**
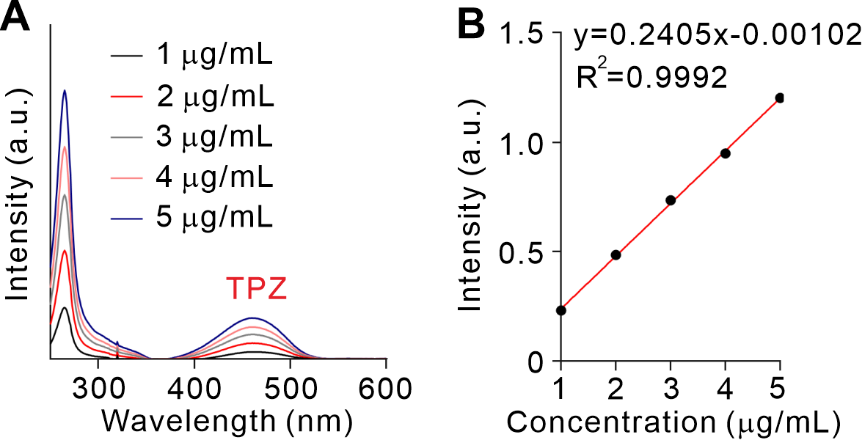
**

**Fig. S2**. (**A**) UV-visible spectra of various concentrations of TPZ solution. (**B**) Linear relation between concentration and corresponding intensity at 265 nm from (**A**).

**
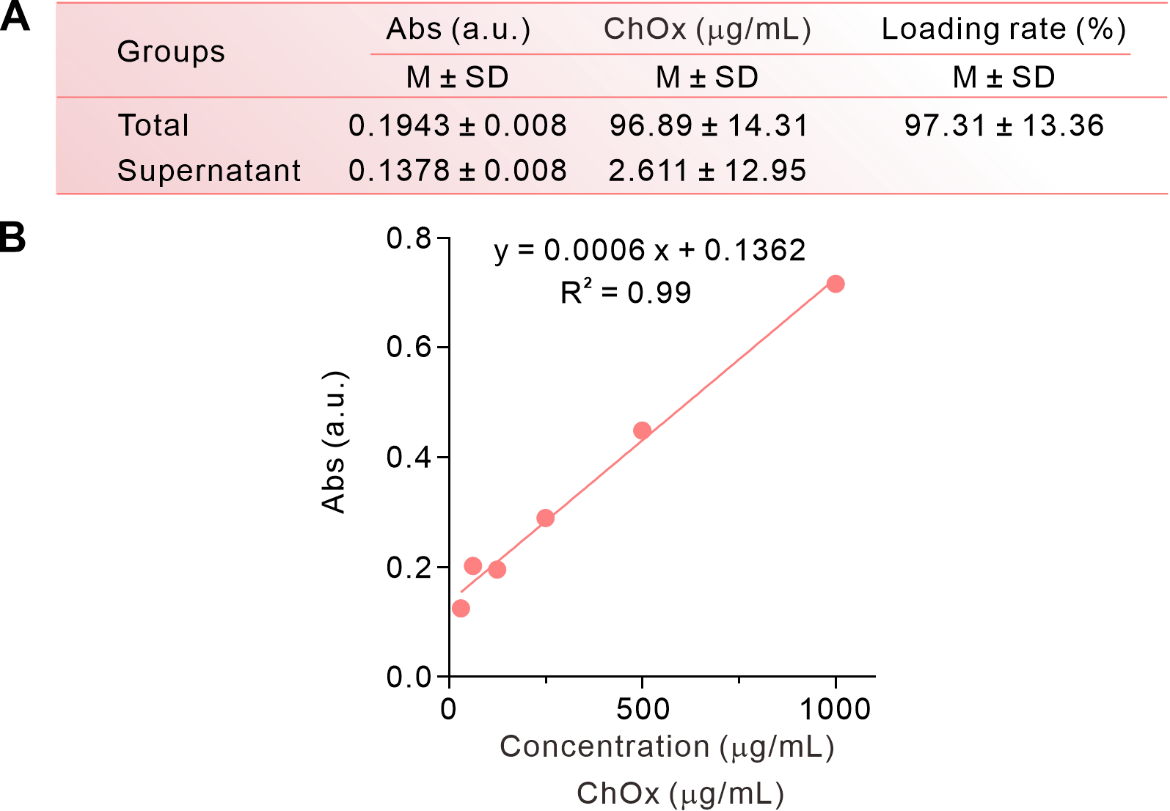
**

**Fig. S3.** (**A**, **B**) The absorbance, calculated concentration, and resulting loading rate of ChOx (**A**) were determined suing the protein standard curve obtained from the BCA protein assay (**B**). Data are presented as mean (M) ± standard deviation (SD), n = 3.

**
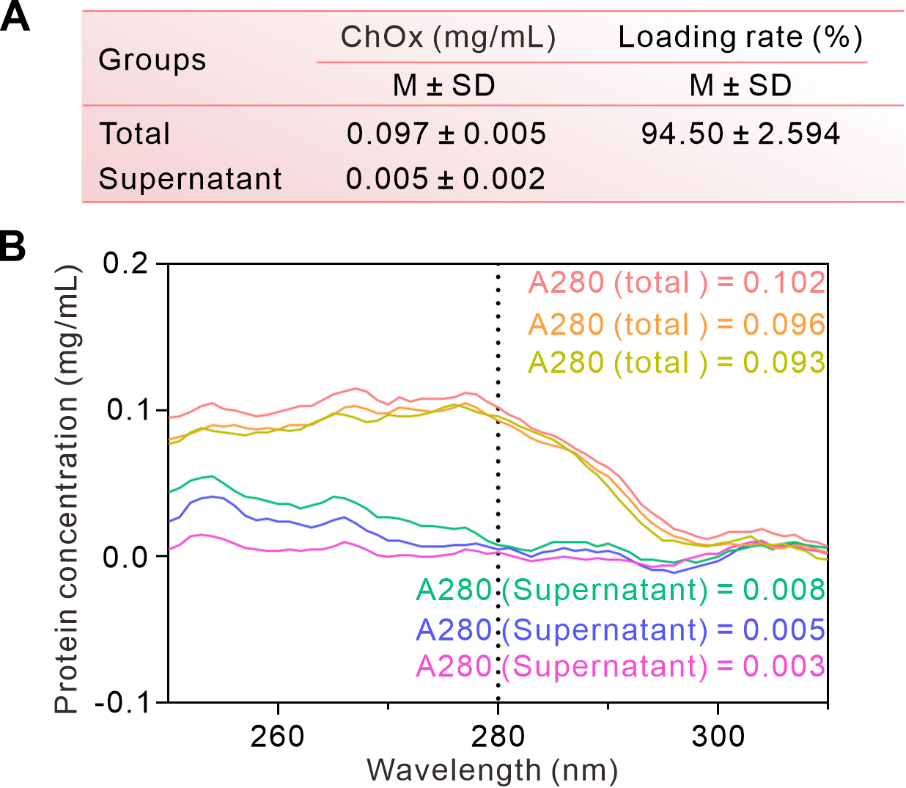
**

**Fig. S4.** (**A**, **B**) The calculated concentration and loading rate of ChOx (**A**) were derived from the protein concentration curve of different groups (total and supernatant) using the nanodrop detection method (**B**), n = 3.

**
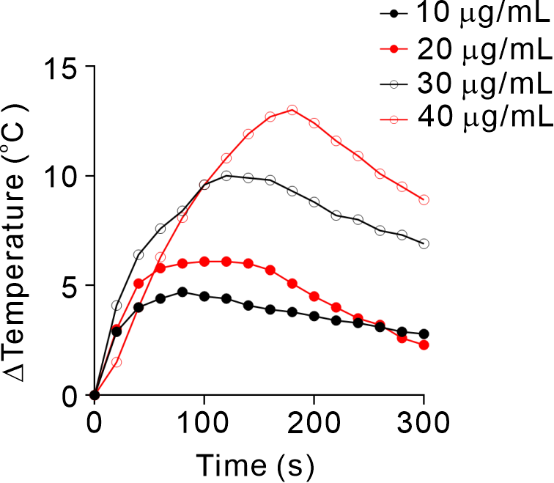
**

**Fig. S5**. Elevated temperature value of various concentrations of free IR806 under 808 nm laser illumination (0.5 W/cm^2^, 5 min).

**
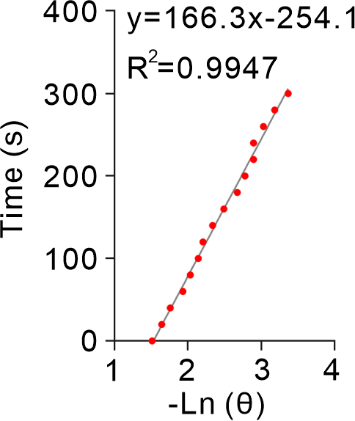
**

**Fig. S6**. Linear fitting results calculated from heating and cooling curves in Figure 1G.

**
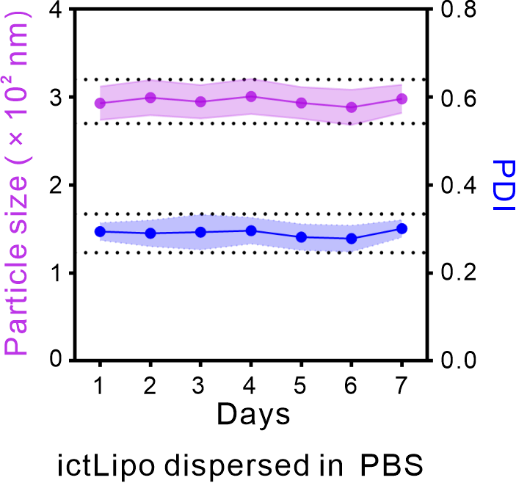
**

**Fig. S7**. Colloidal stability evaluation. Hydrodynamic size and PDI value of ictLipo dispersed in PBS buffer for 7 days.

**
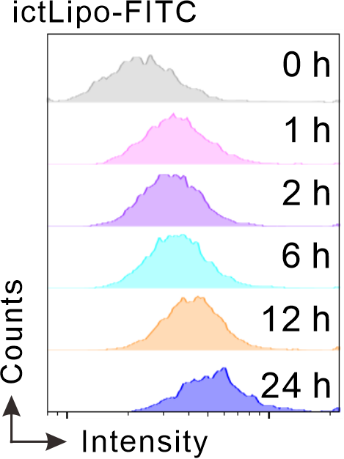
**

**Fig. S8**. Flow cytometric results of cellular uptake of ictLipo in 4T1 cells.

**
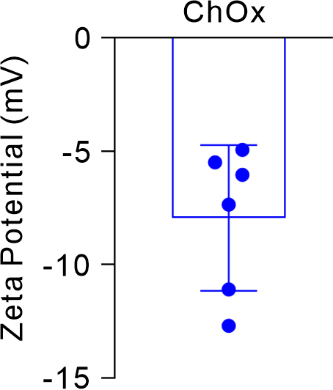
**

**Fig. S9**. Zeta potential values of free ChOx.

**
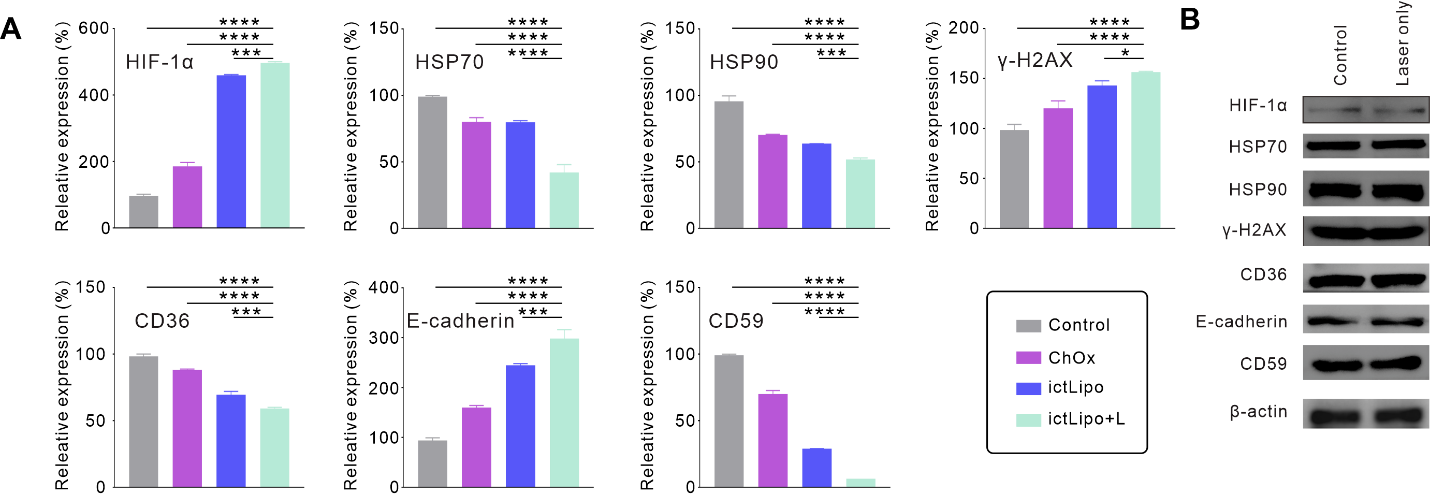
**

**Fig. S10**. The results of the western blotting assay from 4T1 received indicated treatments.

**
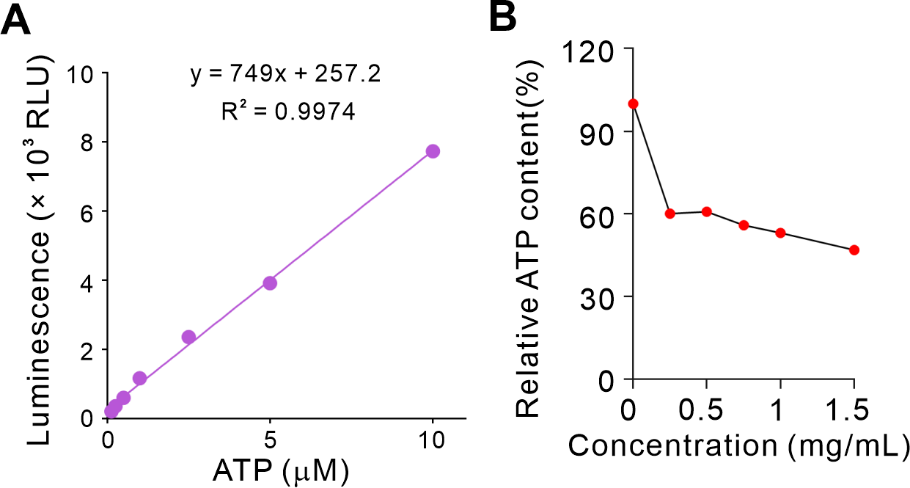
**

**Fig. S11**. (**A**) Standard curve of ATP content measurement kit. (**B**) Intracellular ATP content of 4T1 after incubated with various concentrations of free ChOx.

**
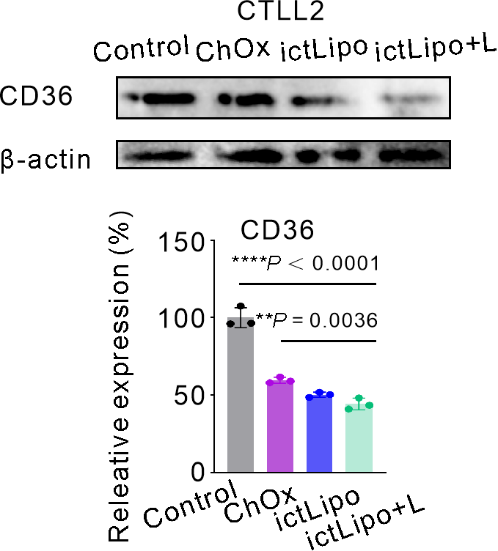
**

**Fig. S12**. Western blotting images and statistical results of CTLL2 cells with indicated treatments.

**
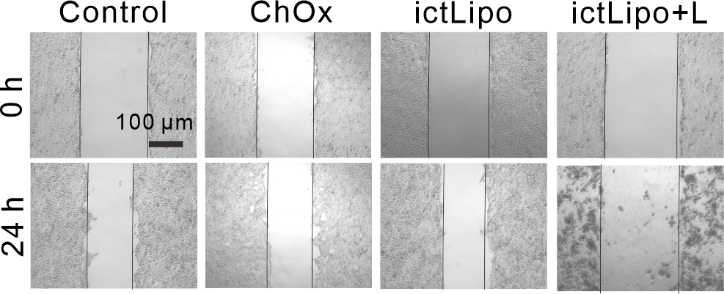
**

**Fig. S13**. Images of scratch assay.

**
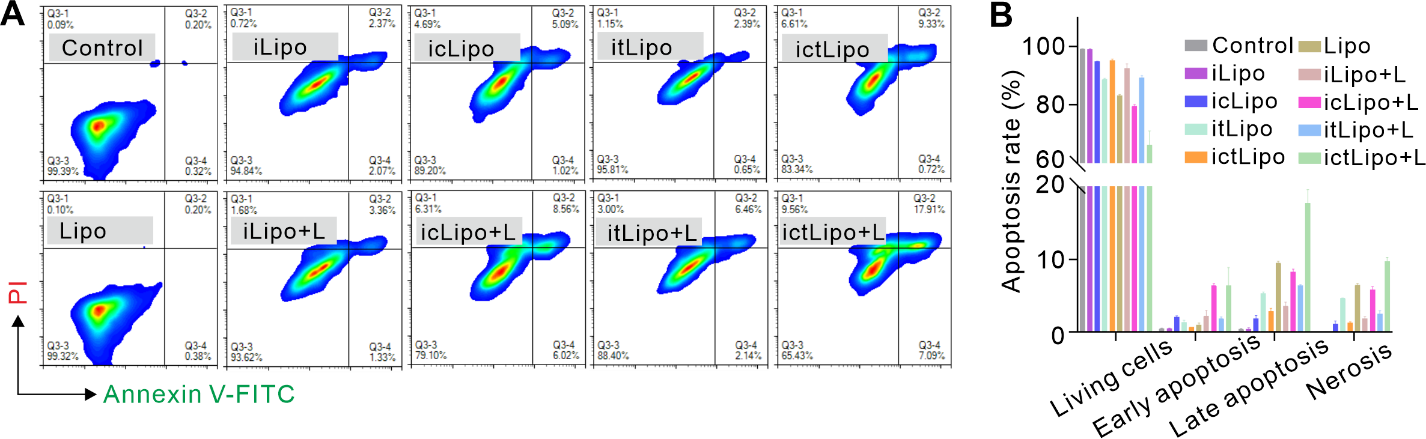
**

**Fig. S14**. (**A, B**) Flow cytometric images of Annexin V-FITC/PI apoptosis results of 4T1 cells treated with various treatments (**A**) and statistical results of apoptosis rate (**B**) from (**A**).

**
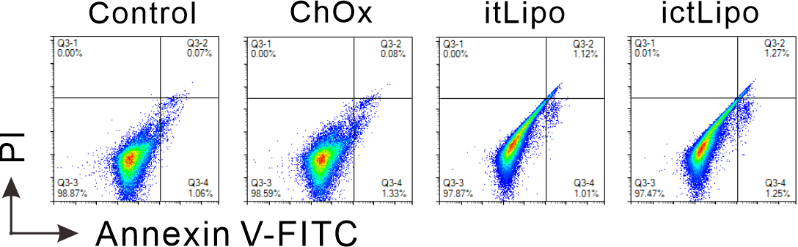
**

**Fig. S15**. Flow cytometry images of Annexin V-FITC/PI of CTLL2 cells with indicated treatments.

**
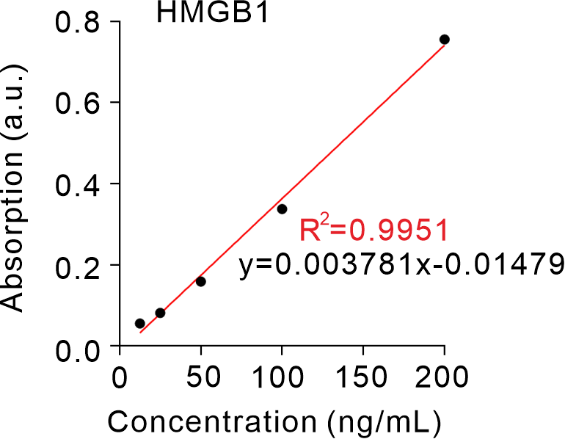
**

**Fig. S16**. Standard curve of Elisa kit to measure HMGB1 content.

**
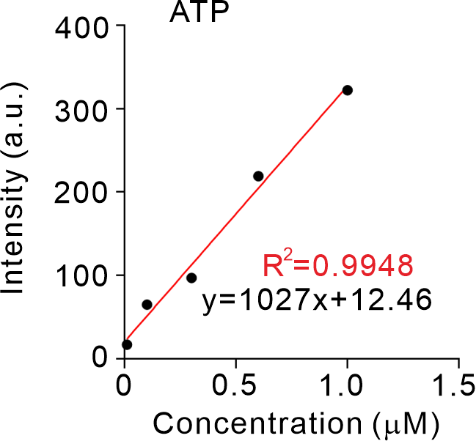
**

**Fig. S17**. Standard curve of ATP kit to measure ATP content.

**
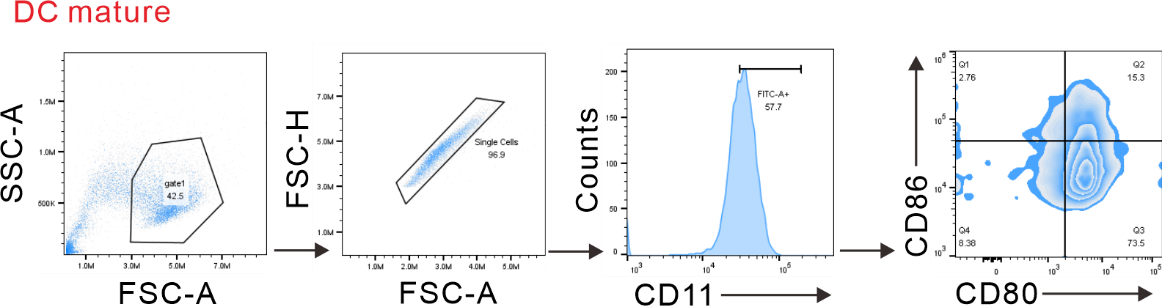
**

**Fig. S18**. Gating strategy of DC mature.

**
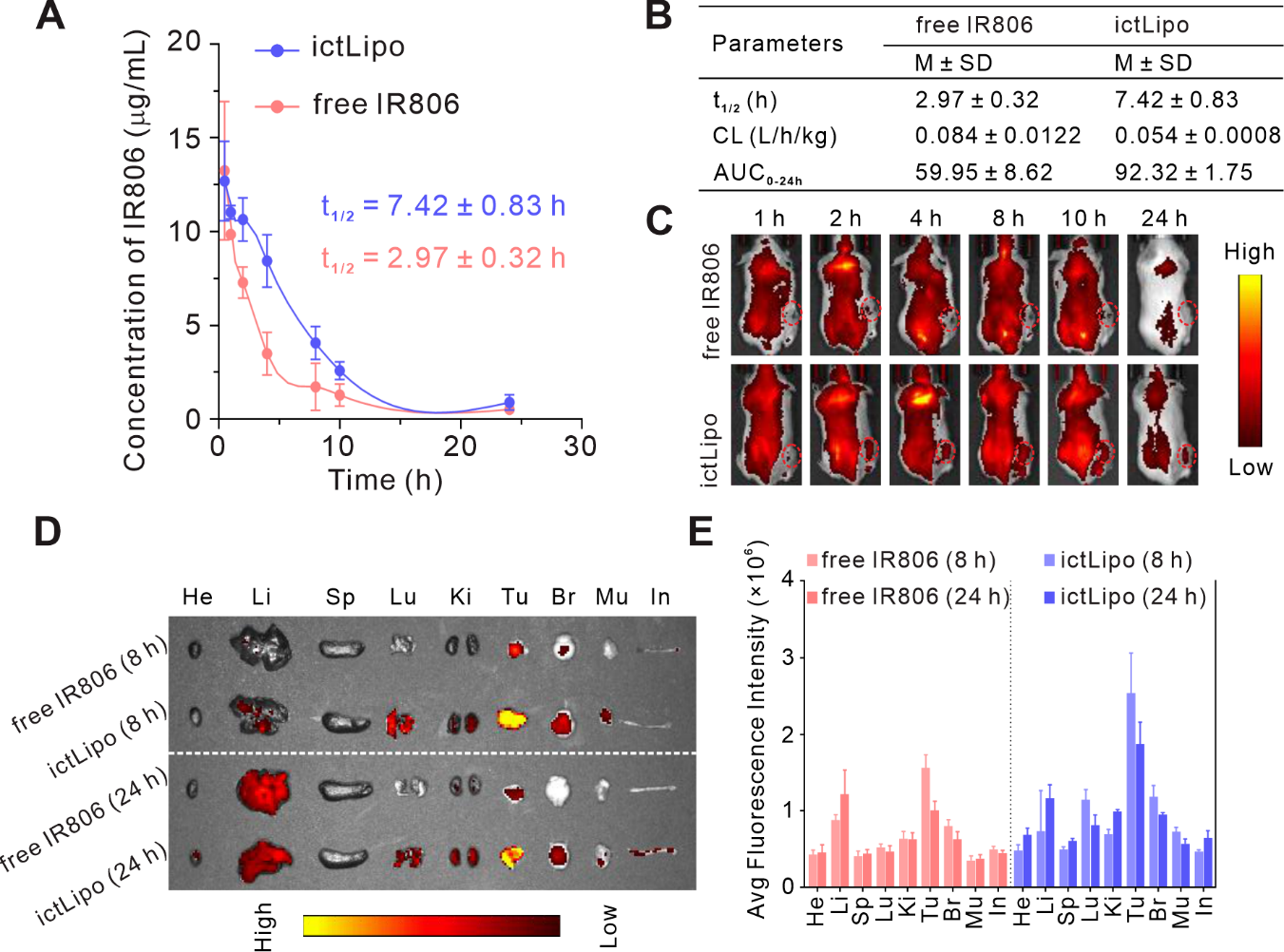
**

**Fig. S19.** (**A**) Plasma concentrations of IR806 over time following intravenous injection of free IR806 and ictLipo at IR806 dose of 5 mg/kg. (**B**) Pharmacokinetic parameters of free IR806 and ictLipo, including half-life (t_1/2_); clearance (CL), and area under the curve (AUC). (**C**) *In vivo* fluorescence images of 4T1 tumor-bearing mice at different time points post-injection of free IR806 and ictLipo. (**D**) *Ex vivo* fluorescence images of tumors (Tu) and major organs, including heart (He), liver (Li), spleen (Sp), lung (Lu), kidney (Ki), brain (Br), muscle (Mu), and intestine (In), collected at 8 h and 24 h post-injection. (**E**) Quantitative analysis of fluorescence in tumors and major organs at 8 and 24 h.

**
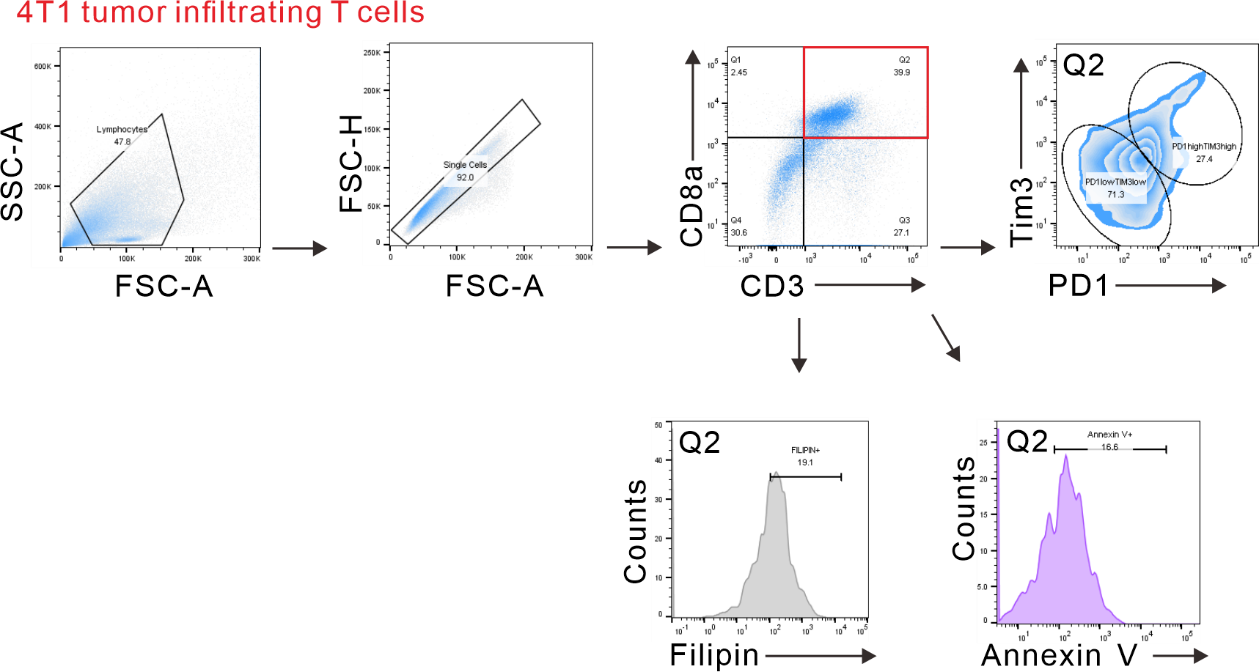
**

**Fig. S20**. Gating strategy of exhausted T cells.

**
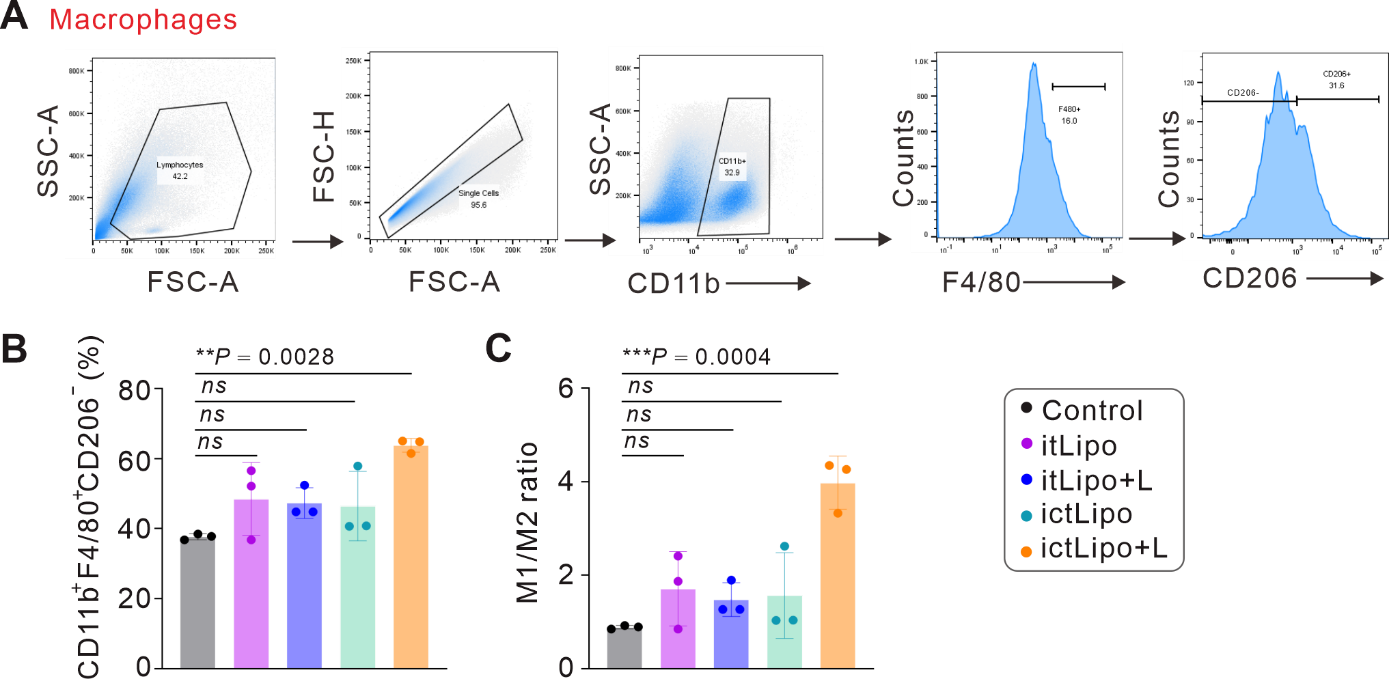
**

**Fig. S21.** (**A**) Gating strategy for identifying macrophages. (**B**) The percentage of M1 macrophages based on statistical analysis. n = 3. (**C**) Ratio of M1 to M2 macrophages. **P < 0.01, ***P < 0.001, and ns: not significant (p > 0.05), analyzed by one-way ANOVA, followed by Dunnett’s multiple comparisons test. Data represent mean ± s.d.

**
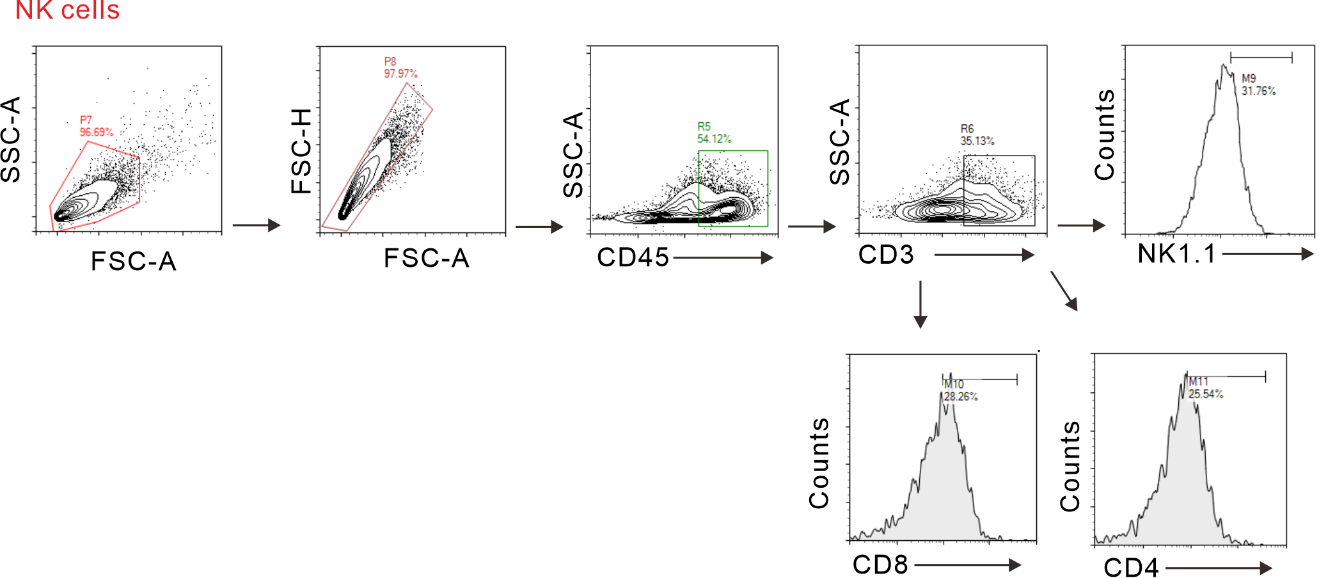
**

**Fig. S22**. Gating strategy of T cells and NK cells.

**
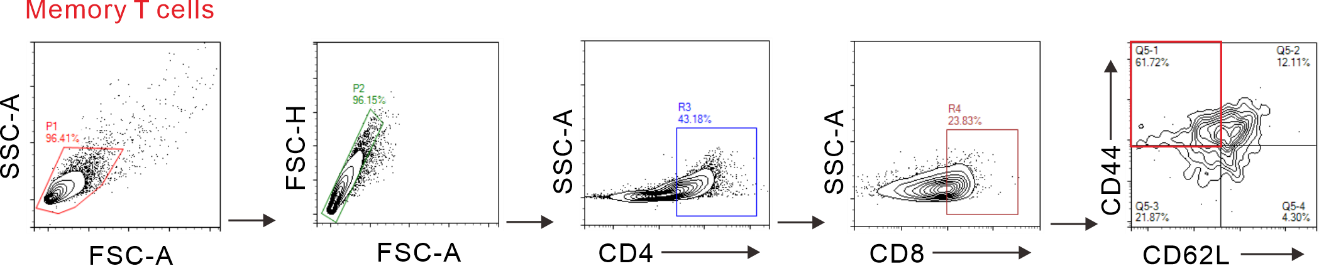
**

**Fig. S23**. Gating strategy of memory T cells.


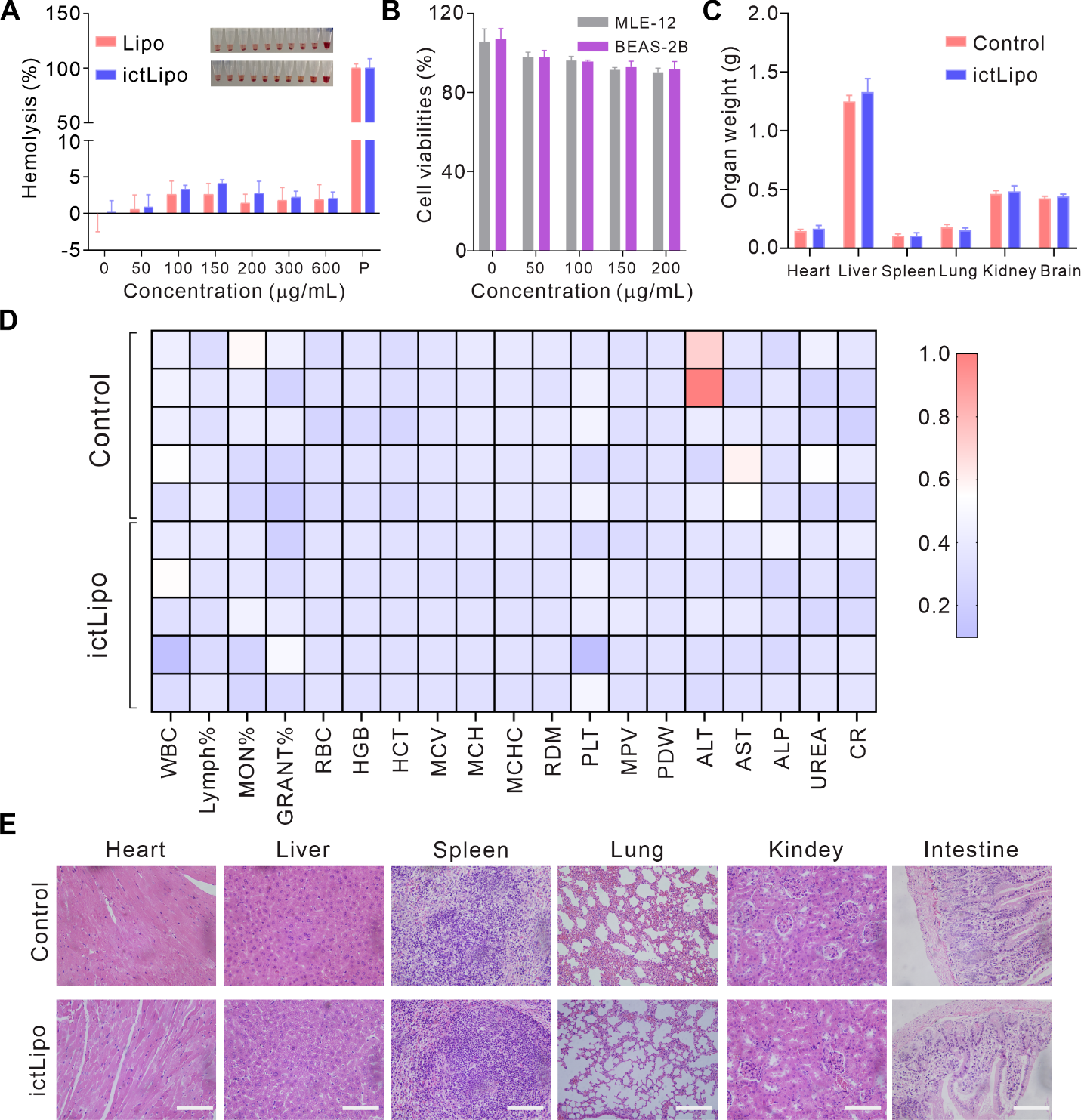


**Fig. S24.** (**A**) Hemolysis rate of Lipo and ictLipo at various concentrations, with corresponding hemolysis images (inset). Triton X-100 (0.5%) was used as a positive control (P). (**B**) Cytotoxicity of ictLipo at different concentrations in MLE-12 (murine lung epithelial-12) and BEAS-2B (human bronchial epithelial cell line) cells after 24 h. (**C**) Changes in organ weights following various treatments. (**D**) Hematological and biochemical parameters. (**E**) H&E staining of major organs. Scale bars are 100 μm.
